# The impact of levothyroxine therapy on the pregnancy, neonatal and childhood outcomes of subclinical hypothyroidism during pregnancy: An updated systematic review, meta-analysis and trial sequential analysis

**DOI:** 10.3389/fendo.2022.964084

**Published:** 2022-08-05

**Authors:** Xue-Feng Jiao, Miao Zhang, Jingjing Chen, Qiang Wei, Linan Zeng, Dan Liu, Chuan Zhang, Hailong Li, Kun Zou, Li Zhang, Lingli Zhang

**Affiliations:** ^1^ Department of Pharmacy, West China Second University Hospital, Sichuan University, Chengdu, China; ^2^ Evidence-Based Pharmacy Center, West China Second University Hospital, Sichuan University, Chengdu, China; ^3^ National Medical Products Administration (NMPA) Key Laboratory for Technical Research on Drug Products *In Vitro* and *In Vivo* Correlation, Chengdu, China; ^4^ Key Laboratory of Birth Defects and Related Diseases of Women and Children, Sichuan University, Ministry of Education, Chengdu, China; ^5^ West China School of Pharmacy, Sichuan University, Chengdu, China; ^6^ Department of Obstetrics and Gynecology, West China Second University Hospital, Sichuan University, Chengdu, China

**Keywords:** subclinical hypothyroidism during pregnancy, levothyroxine, pregnancy outcomes, neonatal outcomes, childhood outcomes

## Abstract

**Background:**

Several systematic reviews and meta-analyses have investigated the effect of levothyroxine (LT4) therapy in pregnant women with subclinical hypothyroidism (SCH). However, all these studies have clinical or methodological problems (such as adopting the old 2011 American Thyroid Association [ATA] diagnostic criteria, directly combining randomized controlled trials [RCTs] and cohort studies for meta-analysis, and so on), and cannot provide accurate and satisfactory results. Thus, we performed this updated systematic review, meta-analysis and trial sequential analysis (TSA) to assess the effect of LT4 therapy in pregnant women with SCH, with the goal of providing more accurate and reliable evidence for clinical practice.

**Methods:**

We searched nine databases from inception to February 2022. The search strategy targeted the RCTs and cohort studies on pregnancy, neonatal and childhood outcomes following LT4 treatment in pregnant women with SCH based on the new 2017 ATA diagnostic criteria. We performed meta-analyses of RCTs and cohort studies separately, and further performed meta-analyses by excluding studies with high risk of bias. TSA was performed to test whether the current evidence was sufficient, and the quality of evidence was evaluated using the GRADE method.

**Results:**

A total of 9 RCTs and 13 cohort studies comprising 11273 pregnant women with SCH were included. There were no statistically significant differences between LT4 group and control group in all primary and secondary outcomes, such as preterm delivery (RR=0.46, 95%CI: 0.19-1.09, *P*=0.08, I^2 =^ 65%), miscarriage (RR=0.36, 95%CI: 0.13-1.03, *P*=0.06, I^2 =^ 38%), gestational hypertension (RR=0.91, 95%CI: 0.58-1.43, *P*=0.69, I^2 =^ 0%), preeclampsia (RR=1.10, 95%CI: 0.61-1.97, *P*=0.76, I^2 =^ 0%), gestational diabetes (RR=0.80, 95%CI: 0.51-1.25, *P*=0.32, I^2 =^ 34%), and so on. TSA showed that the results for all outcomes were insufficient and inconclusive. According to GRADE, the evidences for four outcomes (miscarriage, gestational hypertension, gestational diabetes, and small for gestational age) were rated as moderate quality, while the evidences for the other outcomes were rated as low or very low quality.

**Conclusion:**

Unlike previous systematic reviews and meta-analyses, our study found no evidence of benefit of LT4 therapy on pregnancy, neonatal and childhood outcomes in pregnant women with SCH.

**Systematic Review Registration:**

PROSPERO, https://www.crd.york.ac.uk/prospero/display_record.php?ID=CRD42022321937, identifier CRD42022321937.

## 1 Introduction

Subclinical hypothyroidism (SCH) is the most common thyroid dysfunction during pregnancy, which is defined as elevated thyroid stimulating hormones (TSH) with a normal serum free thyroxine (FT4) level (1). The prevalence of SCH during pregnancy is range from 4% to 13% depending on different cutoff values for TSH (2, 3). Although levothyroxine (LT4) is widely used clinically to treat SCH during pregnancy (4), current guidelines provide very different recommendations in this issue. For example, in the 2019 Chinese Medical Association (CMA) guideline, LT4 therapy is recommended for pregnant women with SCH (5); in the 2017 American Thyroid Association (ATA) guideline, LT4 therapy is strongly recommended for TPOAb-positive women with SCH during pregnancy, and weakly recommended for TPOAb-negative women with SCH during pregnancy (6); whereas in the 2020 American College of Obstetricians and Gynecologists (ACOG) guideline, LT4 therapy is not recommended for pregnant women with SCH, regardless of TPOAb status (7).

In addition, several systematic reviews and meta-analyses have explored the effect of LT4 therapy in pregnant women with SCH. However, all these studies have clinical or methodological problems, and cannot provide accurate and satisfactory results. For example, the majority of these studies adopted the diagnostic criteria for SCH during pregnancy recommended in the 2011 ATA guideline (TSH > 2.5 mIU/L for the first trimester), which was much wider than the new diagnostic criteria recommended in the 2017 ATA guideline (TSH > 4 mIU/L for the first trimester). That is to say, pregnant women with TSH 2.5 - 4.0 mIU/L in the first trimester could not be diagnosed as SCH according to the new 2017 ATA diagnostic criteria, but were considered as SCH cases in these studies. Such misclassification can lead to inaccurate results and less accurate conclusions.

At present, only one systematic review and meta-analysis adopted the new 2017 ATA diagnostic criteria (8), and this study also suffers from a series of problems. First, randomized controlled trials (RCTs) and cohort studies were directly combined in the meta-analysis, which may lead to misleading results. Different types of studies could not be combined for meta-analysis due to methodological heterogeneity (9). Second, some important literature databases, such as the Cochrane Library, the WanFang Database and the VIP Database, were not searched in this study. Thus, there is a strong possibility of missing relevant literature. Third, the outcomes in this study were incomprehensive, lacking some important pregnancy and offspring outcomes. Fourth, the different roles of LT4 in TPOAb-positive and TPOAb-negative women had not been well studied. Fifth, this study neither performed trial sequential analysis (TSA) to test whether the current RCTs and cohort studies had enough statistical power to reach a firm conclusion (10), nor adopted the Grading of Recommendations Assessment, Development, and Evaluation (GRADE) method to evaluate the quality of the current evidence (10). Both TSA and GRADE assessment are important parts of high-quality systematic review and meta-analysis.

To overcome the above problems, we performed an updated systematic review, meta-analysis and TSA to comprehensively assess the impact of LT4 therapy on the pregnancy, neonatal and childhood outcomes of SCH during pregnancy, with the goal of providing more accurate and reliable evidence for clinical practice.

## 2 Methods

This systematic review and meta-analysis was conducted in a pre-specified protocol registered with PROSPERO (CRD42022321937). Our study was reported in line with the preferred reporting items for systematic review and meta-analysis (PRISMA) (11).

### 2.1 Search strategy

We systematically searched literature databases of PubMed, EMbase (Ovid), the Cochrane Library, the China National Knowledge Infrastructure (CNKI), the WanFang Database, the VIP Database and the China Biology Medicine disc from inception to February 2022. In addition, we searched ongoing clinical trial databases, such as “http://www.controlled-trials.com” and “http://clinicaltrials.gov”. The search strategy consisted of the following terms: subclinical, sub-clinical, hypothyroidism, thyroid deficiency, thyroid insufficiency, pregnancy, gestation, thyroxine, levothyroxine, LT4, thyroxine supplementation, thyroxine, synthroid. Moreover, we manually checked the references of included studies. The search strategy in PubMed is shown in **Supplementary Table 1**.

### 2.2 Eligibility criteria

According to the PICOS criteria, the inclusion criteria were as follows: (1) Population: pregnant women who were diagnosed with SCH based on the 2017 ATA guideline (TSH level greater than the upper limit of the pregnancy-specific reference range or [if unavailable] above 4.0 mIU/L in the first trimester); (2) Intervention: thyroxine (including levothyroxine, thyroxine supplementation); (3) Comparison: placebo or no treatment; (4) Outcomes: pregnancy, neonatal and childhood outcomes; (5) Study design: RCT and cohort study. Exclusion criteria were as follows: (1) duplicate publications; (2) the full text was not available; (3) non-Chinese and English literature.

### 2.3 Outcome

The primary outcomes included: (1) pregnancy outcomes: preterm delivery, miscarriage, gestational hypertension, preeclampsia, gestational diabetes; (2) childhood outcomes: childhood Intelligence Quotient (IQ), childhood motor development, childhood behavioral and social competency. The secondary outcomes included: (1) pregnancy outcomes: postpartum hemorrhage, placental abruption, fetal growth restriction, fetal distress, premature rupture of membranes; (2) neonatal outcomes: small for gestational age, low birth weight, neonatal intensive care unit (NICU) admission, neonatal death, respiratory distress syndrome.

### 2.4 Study selection

Two independent reviewers screened the titles and abstracts, then assessed the eligibility based on the full text. Disagreements were resolved by consensus or consultation with a third independent reviewer.

### 2.5 Data extraction

Two independent reviewers extracted relevant information from the included studies using a pre-piloted data extraction form. The extracted information was as follows: (1) general information: title, first author, year of publication, study design, sample size; (2) baseline characteristics of study population: country, age, Body Mass Index (BMI), TSH level, TPOAb status; (3) pregnancy, neonatal and childhood outcomes. Disagreements were resolved by consensus or consultation with a third independent reviewer.

### 2.6 Risk of bias assessment

Two independent reviewers assessed the risk of bias in each included study. Disagreements were resolved by consensus or consultation with a third independent reviewer.

For RCTs, the risk of bias was evaluated using the RoB2 risk of bias assessment tool recommended by the Cochrane Handbook. The RoB2 tool consists of five domains: bias arising from the randomization process, bias due to deviations from intended interventions, bias due to missing outcome data, bias in measurement of the outcome, and bias in selection of the reported result. Moreover, it provides a summary measure of bias for each study categorized as “low risk of bias”, “some concerns (moderate risk of bias)” or “high risk of bias” (12).

For cohort studies, the risk of bias was evaluated using the Newcastle-Ottawa Quality Assessment Scale (NOS). This tool consists of three aspects: the selection of participants, comparability of study groups, and ascertainment of the outcomes of interest. A total score of 7–9 is considered as “low risk of bias”, 4–6 as “moderate risk of bias”, and 0–3 as “high risk of bias” (13).

### 2.7 Quality of evidence

We used GRADE to evaluate the quality of the current evidence of each outcome (14). By considering five limitations (risk of bias, reporting biases, imprecisions, inconsistencies and indirectness), the GRADE method classifies the quality of evidence into four levels, namely high, moderate, low and very low (15).

### 2.8 Statistical analysis

Statistical analysis were conducted by RevMan 5.4. For dichotomous data, we used the relative risk (RR) or odds ratio (OR) with 95% confidence interval (CI) as the effect measure. For continuous data, we used mean difference (MD) with 95% CI as the effect measure. In addition, descriptive analysis was used for outcomes that could not be combined. Heterogeneity was assessed by I-squared (I^2^) test. If heterogeneity was acceptable (I^2^ ≤ 50%), a fixed effect model was used. If heterogeneity was significant (I^2^ > 50%), a random effect model was used.

#### 2.8.1 Main meta-analysis

We performed meta-analyses of RCTs and cohort studies separately. Moreover, to test the influence of poor-quality studies, we also performed meta-analyses by excluding studies with high risk of bias. The Rules for drawing conclusions were as follows (16):

(1) For each outcome, if the meta-analysis result of all included studies was inconsistent with that of studies with low and moderate risk of bias, we drew conclusion based on the meta-analysis result of studies with low and moderate risk of bias. Conversely, if they were consistent, we drew conclusion based on the meta-analysis result of all included studies.

(2) For each outcome, if the evidence strength of RCTs was higher than that of cohort studies, we drew conclusion based on the meta-analysis result of RCTs. Conversely, if the evidence strength of cohort studies was higher, we then drew conclusion based on the meta-analysis result of cohort studies.

#### 2.8.2 Subgroup analysis

To explore heterogeneity due to the effect of TPOAb status on the results, we performed a subgroup analysis stratified by the TPOAb status of the study participants (positive or negative). We followed the same rules for drawing conclusions as in the main meta-analysis.

#### 2.8.3 TSA analysis

TSA was conducted with the TSA viewer version 0.9.5.10 Beta. For each outcome, we used TSA to test whether the current RCTs and cohort studies had enough statistical power to reach a firm conclusion (10). TSA could calculate the required information size (RIS) for meta-analysis, construct both the trial sequential monitoring boundaries for benefit or harm and the futility boundary before reaching RIS (10).

## 3 Results

### 3.1 Search results and study selection

**Figure 1:**
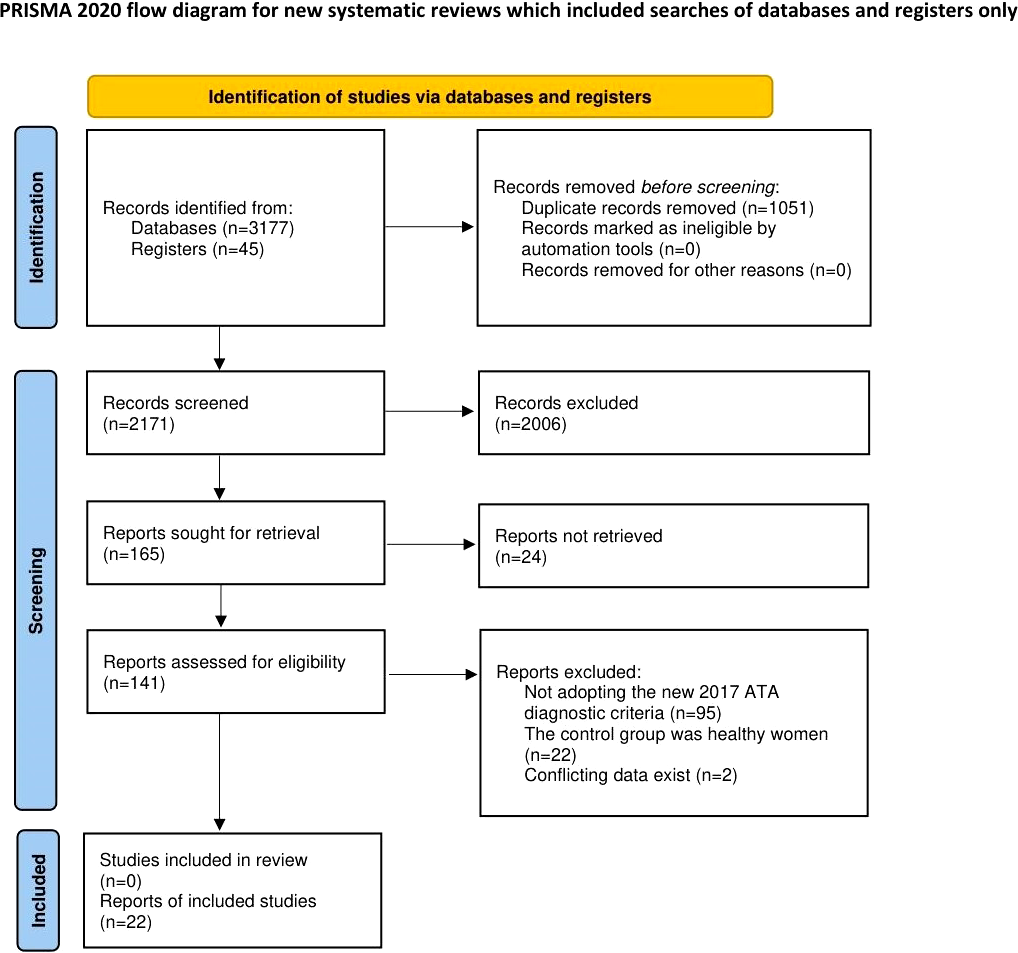
Flow diagram of study selection (PRISMA format).

A total of 3222 articles were identified by the initial search. After selection, 22 studies (17–38) met our inclusion criteria and were included in this systematic review. The literature screening process is shown in **Figure 1**.

### 3.2 Characteristics of included studies

A total of 22 (17–38) studies were included, including 9 RCTs (18–20, 24–26, 30, 33, 37) and 13 cohort studies (17, 21–23, 27–29, 31, 32, 34–36, 38), comprising 11,273 pregnant women with SCH. These studies were conducted in Iran (19, 20), the United States (23, 24), China (17, 22, 25–38), Japan (21) and South Korea (18). Detailed characteristics of the included studies are shown in **Supplementary Table 2**.

### 3.3 Risk of bias assessment

The risk of bias assessment of the included RCTs and cohort studies is shown in **Supplementary Figure 1** and **Supplementary Table 3**, respectively. For RCTs, we determined the following features to be at high risk of bias: randomization process in 1 RCT (30); deviations from intended interventions in 5 RCTs (25, 26, 30, 33, 37); missing outcome data in 5 RCTs (25, 26, 30, 33, 37). Overall, 3 (33%) RCTs (18, 19, 24) were rated at low risk of bias, 1 (11%) RCT (20) was rated at some concerns (moderate risk of bias), and the remaining (56%) RCTs (25, 26, 30, 33, 37) were rated at high risk of bias. For cohort studies, judging by the NOS score, 1 (8%) cohort study (23) was rated at low risk of bias, 4 (31%) cohort studies (17, 21, 22, 31) were rated at moderate risk of bias, and the remaining (61%) studies (27–29, 32, 34–36, 38) were rated at high risk of bias.

### 3.4 Main meta-analysis

#### 3.4.1 Primary outcomes

##### 3.4.1.1 Preterm delivery

A total of 8 RCTs (19, 20, 24–26, 30, 33, 37) and 11 cohort studies (17, 22, 23, 27–29, 32, 34–36, 38) reported preterm delivery.

For RCTs, the meta-analysis of all RCTs indicated that LT4 group had a lower risk of preterm delivery compared with the control group (RR=0.56, 95%CI: 0.43-0.73, *P <* 0.001, I^2 =^ 0%) (19, 20, 24–26, 30, 33, 37). However, when we excluded RCTs with high risk of bias, there was no statistically significant difference between LT4 group and control group in preterm delivery (RR=0.46, 95%CI: 0.19-1.09, *P*=0.080, I^2 =^ 65%) (19, 20, 24) (**Table 1**).

**Table 1:** Meta-analysis results for primary pregnancy outcomes.

Outcome	Included studies	Number of studies	Number of patients	RR or OR (95%CI)	*P*-value	I^2^	Model
Preterm delivery	all RCTs	8	2443	0.56 (0.43, 0.73)	< 0.001	0%	Fix
	RCTs with low and moderate risk of bias	3	895	0.46 (0.19, 1.09)	0.080	65%	Random
	all cohort studies	11	8609	0.57 (0.37, 0.87)	0.009	69%	Random
	cohort studies with low and moderate risk of bias	3	1760	1.21 (0.78, 1.87)	0.390	0%	Fix
Miscarriage	all RCTs	7	2254	0.43 (0.32, 0.57)	< 0.001	0%	Fix
	RCTs with low and moderate risk of bias	2	706	0.36 (0.13, 1.03)	0.060	38%	Fix
	all cohort studies	9	7406	0.55 (0.45, 0.68)	< 0.001	30%	Fix
	cohort studies with low and moderate risk of bias	2	1238	0.47 (0.33, 0.68)	< 0.001	0%	Fix
Gestational hypertension	all RCTs	4	2011	0.63 (0.47, 0.84)	0.002	42%	Fix
	RCTs with low and moderate risk of bias	1	677	0.91 (0.58, 1.43)	0.690	0%	Fix
	all cohort studies	9	8353	0.75 (0.65, 0.87)	< 0.001	0%	Fix
	cohort studies with low and moderate risk of bias	2	1667	0.80 (0.52, 1.22)	0.310	0%	Fix
Preeclampsia	all RCTs	1	677	1.10 (0.61, 1.97)	0.760	0%	Fix
	RCTs with low and moderate risk of bias	1	677	1.10 (0.61, 1.97)	0.760	0%	Fix
	all cohort studies	0	0	NA	NA	NA	NA
	cohort studies with low and moderate risk of bias	0	0	NA	NA	NA	NA
Gestational diabetes	all RCTs	4	1021	0.80 (0.51, 1.25)	0.320	34%	Fix
	RCTs with low and moderate risk of bias	1	677	1.13 (0.65, 1.97)	0.660	0%	Fix
	all cohort studies	9	5004	0.56 (0.36, 0.89)	0.010	75%	Random
	cohort studies with low and moderate risk of bias	2	1667	0.82 (0.25, 2.69)	0.750	93%	Random

RCTs, randomized controlled trials; RR, relative risk; OR, odds ratio; CI, confidence interval; I^2^, statistical heterogeneity; NA, not applicable since no studies were included; According to the pre-defined rules, the meta-analysis results with gray background were used to draw conclusions for each outcome.

For cohort studies, the meta-analysis of all cohort studies indicated that LT4 group had a lower risk of preterm delivery compared with the control group (OR=0.57, 95%CI: 0.37-0.87, *P*=0.009, I^2 =^ 69%) (17, 22, 23, 27–29, 32, 34–36, 38). However, when we excluded cohort studies with high risk of bias, there was no statistically significant difference between LT4 group and control group in preterm delivery (OR=1.21, 95%CI: 0.78-1.87, *P*=0.390, I^2 =^ 0%) (17, 22, 23) (**Table 1**).

**Figure 2:**
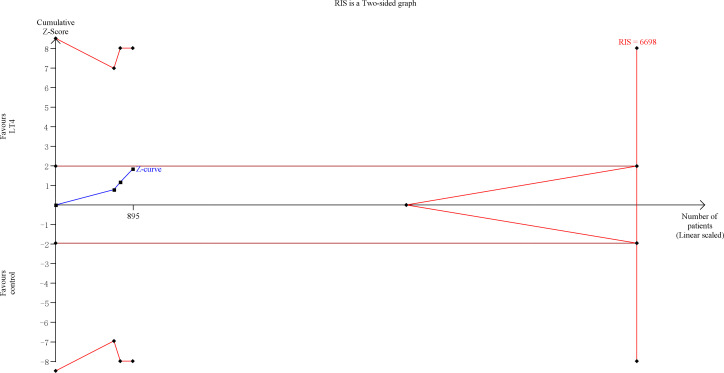
Trial sequential analysis of preterm delivery. The risk of typeIerror was set at 5% with a power of 80%. The variance was calculated from the data obtained from the included trials. The relative risk reduction (RRR) was set at 20%.

TSA showed that the cumulative information size (n=895) was 13% of RIS (n=6698). The cumulative Z-curve did not cross the trial sequential monitoring boundary or the futility boundary, indicating that current evidence was insufficient and inconclusive (**Figure 2**).

The quality of evidence was rated as low for this outcome (**Supplementary Table 5**).

##### 3.4.1.2 Miscarriage

A total of 7 RCTs (18, 24–26, 30, 33, 37) and 9 cohort studies (21, 23, 27, 29, 32, 34–36, 38) reported miscarriage.

For RCTs, the meta-analysis of all RCTs indicated that LT4 group had a lower risk of miscarriage compared with the control group (RR=0.43, 95%CI: 0.32-0.57, *P <* 0.001, I^2 =^ 0%) (18, 24–26, 30, 33, 37). However, when we excluded RCTs with high risk of bias, there was no statistically significant difference between LT4 group and control group in miscarriage (RR=0.36, 95%CI: 0.13-1.03, *P*=0.060, I^2 =^ 38%) (18, 24) (**Table 1**).

For cohort studies, the meta-analysis of all cohort studies indicated that LT4 group had a lower risk of miscarriage compared with the control group (OR=0.55, 95%CI: 0.45-0.68, *P*<0.001, I^2 =^ 30%) (21, 23, 27, 29, 32, 34–36, 38). Moreover, when we excluded cohort studies with high risk of bias, LT4 group still had a lower risk of miscarriage compared with the control group (OR=0.47, 95%CI: 0.33-0.68, *P <* 0.001, I^2 =^ 0%) (21, 23) (**Table 1**).

**Figure 3:**
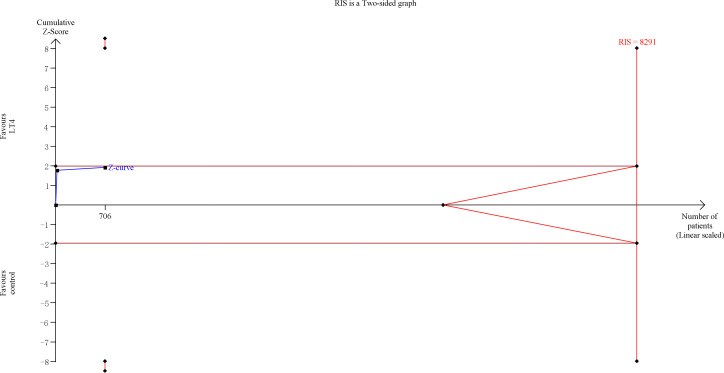
Trial sequential analysis of miscarriage. The risk of typeIerror was set at 5% with a power of 80%. The variance was calculated from the data obtained from the included trials. The relative risk reduction (RRR) was set at 20%.

TSA showed that the cumulative information size (n=1238) was 38% of RIS (n=3289). The cumulative Z-curve did not cross the trial sequential monitoring boundary or the futility boundary, indicating that current evidence was insufficient and inconclusive (**Figure 3**).

The quality of evidence was rated as moderate for this outcome (**Supplementary Table 5**).

##### 3.4.1.3 Gestational hypertension

A total of 4 RCTs (24, 26, 33, 37) and 9 cohort studies (17, 23, 28, 29, 32, 34–36, 38) reported gestational hypertension.

For RCTs, the meta-analysis of all RCTs indicated that LT4 group had a lower risk of gestational hypertension compared with the control group (RR=0.63, 95%CI: 0.47-0.84, *P*=0.002, I^2 =^ 42%) (24, 26, 33, 37). However, when we excluded RCTs with high risk of bias, there was no statistically significant difference between LT4 group and control group in gestational hypertension (RR=0.91, 95%CI: 0.58-1.43, *P*=0.690, I^2 =^ 0%) (24) (**Table 1**).

For cohort studies, the meta-analysis of all cohort studies indicated that LT4 group had a lower risk of gestational hypertension compared with the control group (OR=0.75, 95%CI: 0.65-0.87, *P <* 0.001, I^2 =^ 0%) (17, 23, 28, 29, 32, 34–36, 38). However, when we excluded cohort studies with high risk of bias, there was no statistically significant difference between LT4 group and control group in gestational hypertension (OR=0.80, 95%CI: 0.52-1.22, *P*=0.310, I^2 =^ 0%) (17, 23) (**Table 1**).

**Figure 4:**
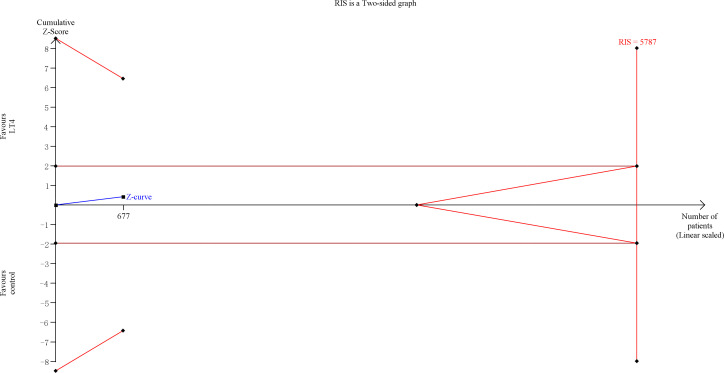
Trial sequential analysis of gestational hypertension. The risk of type I error was set at 5% with a power of 80%. The variance was calculated from the data obtained from the included trials. The relative risk reduction (RRR) was set at 20%.

TSA showed that the cumulative information size (n=677) was 12% of RIS (n=5787). The cumulative Z-curve did not cross the trial sequential monitoring boundary or the futility boundary, indicating that current evidence was insufficient and inconclusive (**Figure 4**).

The quality of evidence was rated as moderate for this outcome (**Supplementary Table 5**).

##### 3.4.1.4 Preeclampsia

Only 1 RCT (24) reported preeclampsia. This study showed that there was no statistically significant difference between LT4 group and control group in preeclampsia (RR=1.10, 95%CI: 0.61-1.97, *P*=0.760, I^2 =^ 0%) (24) (**Table 1**).

**Figure 5:**
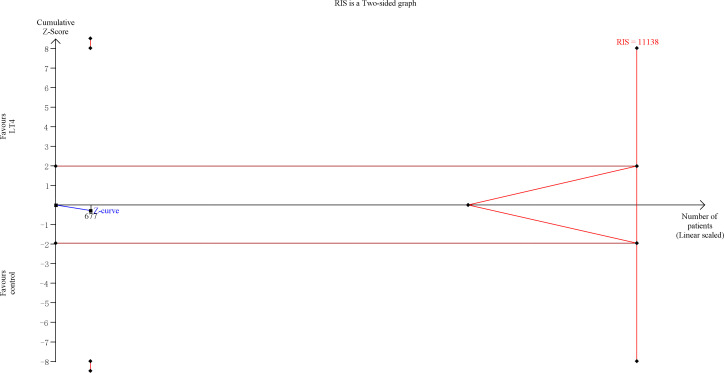
Trial sequential analysis of preeclampsia. The risk of type I error was set at 5% with a power of 80%. The variance was calculated from the data obtained from the included trials. The relative risk reduction (RRR) was set at 20%.

TSA showed that the cumulative information size (n=677) was 6% of RIS (n=11,138). The cumulative Z-curve did not cross the trial sequential monitoring boundary or the futility boundary, indicating that current evidence was insufficient and inconclusive (**Figure 5**).

The quality of evidence was rated as low for this outcome (**Supplementary Table 5**).

##### 3.4.1.5 Gestational diabetes

A total of 4 RCTs (24–26, 37) and 9 cohort studies (17, 23, 27–29, 32, 34–36) reported gestational diabetes.

For RCTs, the meta-analysis of all RCTs showed that there was no statistically significant different between LT4 group and control group in gestational diabetes (RR=0.80, 95%CI: 0.51-1.25, *P*=0.320, I^2 =^ 34%) (24–26, 37). Moreover, when we excluded RCTs with high risk of bias, there was no statistically significant different between LT4 group and control group in gestational diabetes (RR=1.13, 95%CI: 0.65-1.97, *P*=0.660, I^2 =^ 0%) (24) (**Table 1**).

For cohort studies, the meta-analysis of all cohort studies indicated that LT4 group had a lower risk of gestational diabetes compared with the control group (OR=0.56, 95%CI: 0.36-0.89, *P*=0.010, I^2 =^ 75%) (17, 23, 27–29, 32, 34–36). However, when we excluded cohort studies with high risk of bias, there was no statistically significant difference between LT4 group and control group in gestational diabetes (OR=0.82, 95%CI: 0.25-2.69, *P*=0.750, I^2 =^ 93%) (17, 23) (**Table 1**).

**Figure 6:**
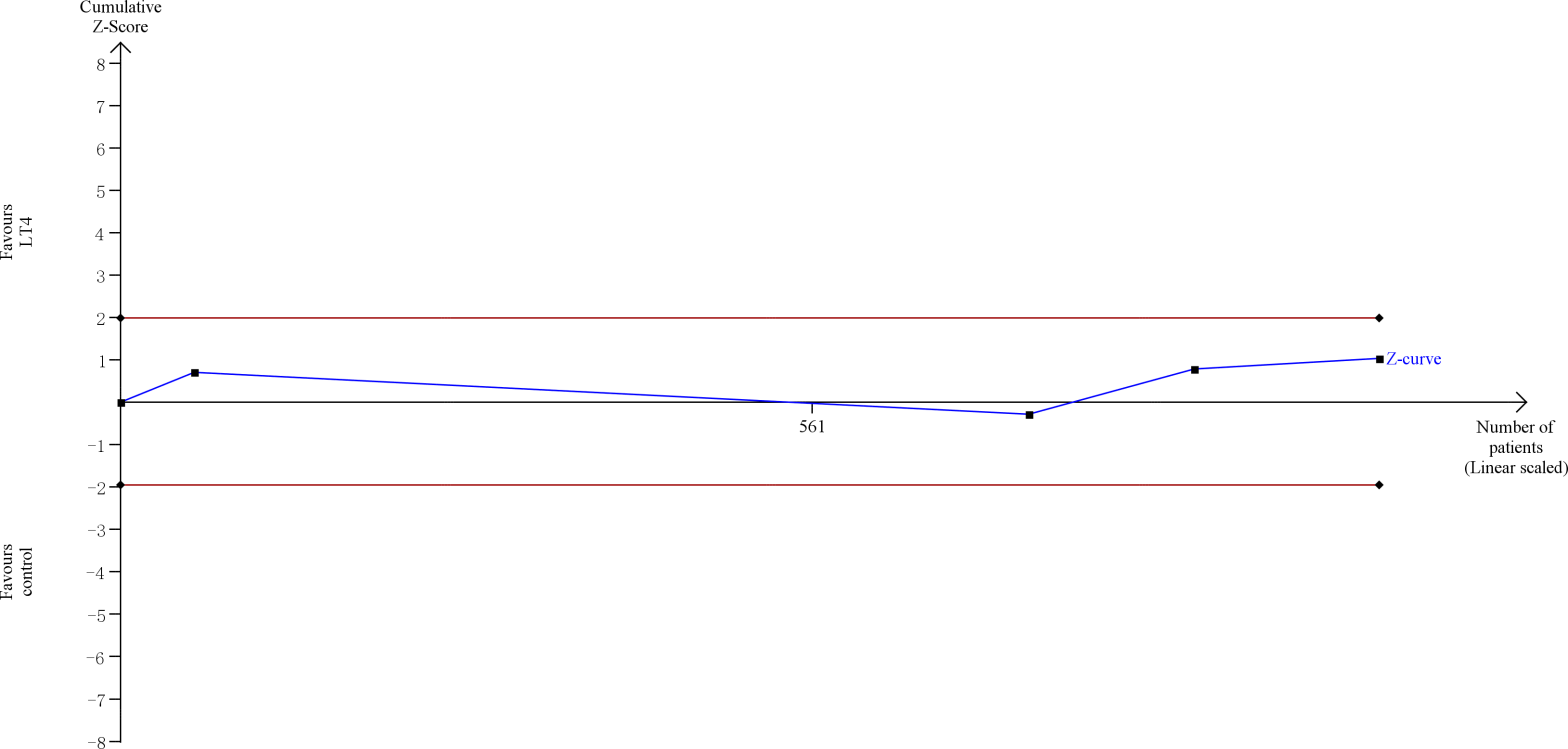
Trial sequential analysis of gestational diabetes. The risk of type I error was set at 5% with a power of 80%. The variance was calculated from the data obtained from the included trials. The relative risk reduction (RRR) was set at 20%.

TSA showed that the cumulative information size (n=1021) was 3% of RIS (n=35,044). The cumulative Z-curve did not cross the trial sequential monitoring boundary or the futility boundary, indicating that current evidence was insufficient and inconclusive (**Figure 6**).

The quality of evidence was rated as moderate for this outcome (**Supplementary Table 5**).

##### 3.4.1.6 Childhood IQ

Only 1 RCT (24) and 1 cohort study (31) reported childhood IQ. The RCT indicated that there was no statistically significant difference between LT4 group and control group in childhood IQ score at 5 years (median 97, 95%CI: 95-99 vs median 95, 95%CI: 93-97; *P*=0.89) *(* 24). Similarly, the cohort study indicated that there was no statistically significant difference between LT4 group and control group in children IQ score at 20-30 months (115.32 ± 13.01 vs 112.78 ± 12.39, *P*=0.27) *(* 31).

##### 3.4.1.7 Childhood motor development

Only 1 cohort study (31) reported childhood motor development. This study indicated that there was no statistically significant difference between LT4 group and control group in childhood motor development score at 20-30 months (115.23 ± 13.41 vs 113.13 ± 10.06, *P*=0.30) *(* 31).

##### 3.4.1.8 Childhood behavioral and social competency

Only 1 RCT (24) reported childhood behavioral and social competency. This study indicated that there were no statistically significant differences between LT4 group and control group in child behavior checklist T-Score at 3 years (median 46, 95%CI: 45-48 vs median 46, 95%CI: 45-48; *P*=0.99) and at 5 years (median 44, 95%CI: 43-46 vs median 44, 95%CI: 42-46; *P*=0.96) *(* 24).

#### 3.4.2 Secondary outcomes

The meta-analyses of RCTs showed that there were no statistically significant differences between LT4 group and control group in placental abruption (RR=0.23, 95%CI: 0.04-1.35, *P*=0.100, I^2 =^ 0%) (24, 37), fetal growth restriction (RR=0.50, 95%CI: 0.16-1.58, *P*=0.240, I^2 =^ 0%) (37), fetal distress (RR=0.86, 95%CI: 0.30-2.42, *P*=0.770, I^2 =^ 0%) (37), low birth weight (RR=0.45, 95%CI: 0.19-1.08, *P*=0.070, I^2 =^ 54%) (33, 37), small for gestational age (RR=1.22, 95%CI: 0.75-1.98, *P*=0.430, I^2 =^ 0%) (24), NICU admission (RR=0.31, 95%CI: 0.01-12.16, *P*=0.530, I^2 =^ 85%) (20, 24), neonatal death (RR=0.33, 95%CI: 0.01-8.13, *P*=0.500, I^2 =^ 0%) (24), or respiratory distress syndrome (RR=1.50, 95%CI: 0.54-4.16, *P*=0.440, I^2 =^ 0%) (24) (**Supplementary Table 4**).

The meta-analyses of cohort studies also showed that there were no statistically significant differences between LT4 group and control group in postpartum hemorrhage (OR=1.05, 95%CI: 0.42-2.66, *P*=0.910, I^2 =^ 0%) (17, 22), placental abruption (OR=1.13, 95%CI: 0.73-1.74, *P*=0.950, I^2 =^ 0%) (27, 28, 32, 38), fetal growth restriction (OR=0.79, 95%CI: 0.60-1.03, *P*=0.080, I^2 =^ 51%) (23, 32, 36, 38), fetal distress (RR=0.78, 95%CI: 0.59-1.04, *P*=0.090, I^2 =^ 0%) (17), premature rupture of membranes (OR=0.73, 95%CI: 0.51-1.05, *P*=0.090, I^2 =^ 0%) (17, 28), low birth weight (OR=1.47, 95%CI: 0.53-4.11, *P*=0.460, I^2 =^ 0%) (17, 22) or small for gestational age (OR=2.14, 95%CI: 0.24-18.79, *P*=0.490, I^2 =^ 0%) (27) (**Supplementary Table 4**).

TSA showed that the current evidences for postpartum hemorrhage, placental abruption, fetal growth restriction, fetal distress, premature rupture of membranes, low birth weight, small for gestational age, NICU admission, neonatal death and respiratory distress syndrome were insufficient and inconclusive (**Supplementary Figure 2–11**).

According to GRADE, the quality of evidence was rated as moderate for small for gestational age; rated as low for placental abruption, neonatal death, and respiratory distress syndrome; rated as very low for postpartum hemorrhage, fetal growth restriction, fetal distress, premature rupture of membranes, low birth weight, and NICU admission (**Supplementary Table 5**).

### 3.5 Subgroup analysis

#### 3.5.1 TPOAb-positive

The meta-analysis of RCTs indicated that LT4 group had lower risks of preterm delivery (RR=0.18, 95%CI: 0.04-0.76, *P*=0.020, I^2 =^ 0%) (20) and NICU admission (RR=0.04, 95%CI: 0.00-0.70, *P*=0.030, I^2 =^ 0%) (20) compared with the control group (**Table 2**).

**Table 2:** Meta-analysis results of the TPOAb-positive subgroup.

Outcome	Included studies	Number of studies	Number of patients	RR or OR (95%CI)	*P*-value	I^2^	Model
Preterm delivery	all RCTs	1	72	0.18 (0.04, 0.76)	0.020	0%	Fix
	RCTs with low and moderate risk of bias	1	72	0.18 (0.04, 0.76)	0.020	0%	Fix
	all cohort studies	3	3411	0.32 (0.23, 0.43)	< 0.001	0%	Fix
	cohort studies with low and moderate risk of bias	0	0	NA	NA	NA	NA
Miscarriage	all RCTs	0	0	NA	NA	NA	NA
	RCTs with low and moderate risk of bias	0	0	NA	NA	NA	NA
	all cohort studies	3	3411	0.32 (0.23, 0.43)	< 0.001	0%	Fix
	cohort studies with low and moderate risk of bias	0	0	NA	NA	NA	NA
Gestational hypertension	all RCTs	0	0	NA	NA	NA	NA
	RCTs with low and moderate risk of bias	0	0	NA	NA	NA	NA
	all cohort studies	3	3411	0.29 (0.17, 0.50)	< 0.001	69%	Random
	cohort studies with low and moderate risk of bias	0	0	NA	NA	NA	NA
Gestational diabetes	all RCTs	0	0	NA	NA	NA	NA
	RCTs with low and moderate risk of bias	0	0	NA	NA	NA	NA
	all cohort studies	2	1298	0.43 (0.25, 0.76)	0.004	0%	Fix
	cohort studies with low and moderate risk of bias	0	0	NA	NA	NA	NA
Placental abruption	all RCTs	0	0	NA	NA	NA	NA
	RCTs with low and moderate risk of bias	0	0	NA	NA	NA	NA
	all cohort studies	2	3335	0.84 (0.34, 2.06)	0.710	0%	Fix
	cohort studies with low and moderate risk of bias	0	0	NA	NA	NA	NA
Fetal growth restriction	all RCTs	0	0	NA	NA	NA	NA
	RCTs with low and moderate risk of bias	0	0	NA	NA	NA	NA
	all cohort studies	3	3411	0.38 (0.30, 0.49)	< 0.001	0%	Fix
	cohort studies with low and moderate risk of bias	0	0	NA	NA	NA	NA
Fetal distress	all RCTs	0	0	NA	NA	NA	NA
	RCTs with low and moderate risk of bias	0	0	NA	NA	NA	NA
	all cohort studies	2	3335	1.12 (0.52, 2.38)	0.770	0%	Fix
	cohort studies with low and moderate risk of bias	0	0	NA	NA	NA	NA
Low birth weight	all RCTs	0	0	NA	NA	NA	NA
	RCTs with low and moderate risk of bias	0	0	NA	NA	NA	NA
	all cohort studies	3	3411	0.40 (0.31, 0.53)	< 0.001	0%	Fix
	cohort studies with low and moderate risk of bias	0	0	NA	NA	NA	NA
NICU admission	all RCTs	1	72	0.04 (0.00, 0.70)	0.030	0%	Fix
	RCTs with low and moderate risk of bias	1	72	0.04 (0.00, 0.70)	0.030	0%	Fix
	all cohort studies	0	0	NA	NA	NA	NA
	cohort studies with low and moderate risk of bias	0	0	NA	NA	NA	NA

TPOAb, thyroid peroxidase antibody; RCTs, randomized controlled trials; RR, risk ratio; OR, odds ratio; CI, confidence interval; NICU, neonatal intensive care unit; I^2^, statistical heterogeneity; NA, not applicable since no studies were included; According to the pre-defined rules, the meta-analysis results with gray background were used to draw conclusions for each outcome.

The meta-analyses of cohort studies indicated that LT4 group had lower risks of preterm delivery (OR=0.32, 95%CI: 0.23-0.43, *P* < 0.001, I^2 =^ 0%) (32, 36, 38), miscarriage (OR=0.32, 95%CI: 0.23-0.43, *P* < 0.001, I^2 =^ 0%) (32, 36, 38), gestational hypertension (OR=0.29, 95%CI: 0.17-0.50, *P* < 0.001, I^2 =^ 69%) (32, 36, 38), gestational diabetes (OR=0.43, 95%CI: 0.25-0.76, *P*=0.004, I^2 =^ 0%) (32, 36), fetal growth restriction (OR=0.38, 95%CI: 0.30-0.49, *P* < 0.001, I^2 =^ 0%) (32, 36, 38), and low birth weight (OR=0.40, 95%CI: 0.31-0.53, *P* < 0.001, I^2 =^ 0%) (32, 36, 38) compared with the control group. However, there were no statistically significant differences between LT4 group and control group in placental abruption (OR=0.84, 95%CI: 0.34-2.06, *P*=0.710, I^2 =^ 0%) (32, 38) or fetal distress (OR=1.12, 95%CI: 0.52-2.38, *P*=0.770, I^2 =^ 0%) (32, 38) (**Table 2**).

TSA showed that the current evidences for miscarriage, gestational hypertension, fetal growth restriction, and low birth weight were sufficient to reach firm conclusions, whereas the current evidences for preterm delivery, gestational diabetes, placental abruption, fetal distress, and NICU admission were insufficient and inconclusive (**Supplementary Figure 12–20**).

According to GRADE, the quality of evidence was rated as low for preterm delivery and NICU admission; rated as very low for miscarriage, gestational hypertension, gestational diabetes, placental abruption, fetal growth restriction, fetal distress, and low birth weight (**Supplementary Table 6**).

#### 3.5.2 TPOAb-negative

The meta-analyses of RCTs indicated that LT4 group had lower risks of preterm delivery (RR=0.49, 95%CI: 0.34-0.69, *P* < 0.001, I^2 =^ 0%) (19, 26, 33, 37), miscarriage (RR=0.43, 95%CI: 0.32-0.59, *P* < 0.001, I^2 =^ 6%) (26, 33, 37), gestational hypertension (RR=0.49, 95%CI: 0.34-0.72, *P* < 0.001, I^2 =^ 0%) (26, 33, 37) and gestational diabetes (RR=0.38, 95%CI: 0.15-0.96, *P*=0.040, I^2 =^ 0%) (26, 37) compared with the control group. However, there were no statistically significant differences between LT4 group and control group in placental abruption (RR=0.33, 95%CI: 0.01-8.04, *P*=0.500, I^2 =^ 0%) (37), fetal growth restriction (RR=0.50, 95%CI: 0.16-1.58, *P*=0.240, I^2 =^ 0%) (37), fetal distress (RR=0.86, 95%CI: 0.30-2.42, *P*=0.770, I^2 =^ 0%) (37) or low birth weight (RR=0.45, 95%CI: 0.19-1.08, *P*=0.070, I^2 =^ 54%) (33, 37) (**Table 3**).

**Table 3:** Meta-analysis results of the TPOAb-negative subgroup.

Outcome	Included studies	Number of studies	Number of patients	RR or OR (95%CI)	*P*-value	I^2^	Model
Preterm delivery	all RCTs	4	1480	0.49 (0.34, 0.69)	< 0.001	0%	Fix
	RCTs with low and moderate risk of bias	1	146	0.37 (0.14, 0.95)	0.040	0%	Fix
	all cohort studies	4	2993	0.77 (0.30, 1.96)	0.580	85%	Random
	cohort studies with low and moderate risk of bias	1	93	2.70 (0.47, 15.55)	0.270	0%	Fix
Miscarriage	all RCTs	3	1334	0.43 (0.32, 0.59)	< 0.001	6%	Fix
	RCTs with low and moderate risk of bias	0	0	NA	NA	NA	NA
	all cohort studies	2	2219	0.97 (0.59, 1.59)	0.900	0%	Fix
	cohort studies with low and moderate risk of bias	0	0	NA	NA	NA	NA
Gestational hypertension	all RCTs	3	1344	0.49 (0.34, 0.72)	< 0.001	0%	Fix
	RCTs with low and moderate risk of bias	0	0	NA	NA	NA	NA
	all cohort studies	3	2900	0.84 (0.62, 1.13)	0.250	2%	Fix
	cohort studies with low and moderate risk of bias	0	0	NA	NA	NA	NA
Gestational diabetes	all RCTs	2	194	0.38 (0.15, 0.96)	0.040	0%	Fix
	RCTs with low and moderate risk of bias	0	0	NA	NA	NA	NA
	all cohort studies	2	1501	0.46 (0.26, 0.83)	0.010	55%	Random
	cohort studies with low and moderate risk of bias	0	0	NA	NA	NA	NA
Postpartum hemorrhage	all RCTs	0	0	NA	NA	NA	NA
	RCTs with low and moderate risk of bias	0	0	NA	NA	NA	NA
	all cohort studies	1	93	0.63 (0.05, 7.14)	0.710	0%	Fix
	cohort studies with low and moderate risk of bias	1	93	0.63 (0.05, 7.14)	0.710	0%	Fix
Placental abruption	all RCTs	1	134	0.33 (0.01, 8.04)	0.500	0%	Fix
	RCTs with low and moderate risk of bias	0	0	NA	NA	NA	NA
	all cohort studies	3	2900	1.23 (0.70, 2.17)	0.470	0%	Fix
	cohort studies with low and moderate risk of bias	0	0	NA	NA	NA	NA
Fetal growth restriction	all RCTs	1	134	0.50 (0.16, 1.58)	0.240	0%	Fix
	RCTs with low and moderate risk of bias	0	0	NA	NA	NA	NA
	all cohort studies	2	2219	0.99 (0.72, 1.37)	0.970	0%	Fix
	cohort studies with low and moderate risk of bias	0	0	NA	NA	NA	NA
Fetal distress	all RCTs	1	134	0.86 (0.30, 2.42)	0.770	0%	Fix
	RCTs with low and moderate risk of bias	0	0	NA	NA	NA	NA
	all cohort studies	3	2900	0.66 (0.44, 0.99)	0.040	48%	Fix
	cohort studies with low and moderate risk of bias	0	0	NA	NA	NA	NA
Premature rupture of membranes	all RCTs	0	0	NA	NA	NA	NA
	RCTs with low and moderate risk of bias	0	0	NA	NA	NA	NA
	all cohort studies	1	681	0.80 (0.47, 1.38)	0.420	0%	Fix
	cohort studies with low and moderate risk of bias	0	0	NA	NA	NA	NA
Low birth weight	all RCTs	2	1274	0.45 (0.19, 1.08)	0.070	54%	Random
	RCTs with low and moderate risk of bias	0	0	NA	NA	NA	NA
	all cohort studies	4	2993	0.99 (0.72, 1.37)	0.970	0%	Fix
	cohort studies with low and moderate risk of bias	1	93	3.89 (0.15, 97.99)	0.410	0%	Fix

TPOAb, thyroid peroxidase antibody; RCTs, randomized controlled trials; RR, risk ratio; OR, odds ratio; CI, confidence interval; I^2^, statistical heterogeneity; NA, not applicable since no studies were included; According to the pre-defined rules, the meta-analysis results with gray background were used to draw conclusions for each outcome.

The meta-analyses of cohort studies indicated that LT4 group had lower risks of gestational diabetes (OR=0.46, 95%CI: 0.26-0.83, *P*=0.010, I^2 =^ 55%) (28, 32) and fetal distress (OR=0.66, 95%CI: 0.44-0.99, *P*=0.040, I^2 =^ 48%) (28, 32, 38) compared with the control group. However, there were no statistically significant differences between LT4 group and control group in preterm delivery (OR=0.77, 95%CI: 0.30-1.96, *P*=0.580, I^2 =^ 85%) (22, 28, 32, 38), miscarriage (OR=0.97, 95%CI: 0.59-1.59, *P*=0.900, I^2 =^ 0%) (26, 33, 37), gestational hypertension (OR=0.84, 95%CI: 0.62-1.13, *P*=0.250, I^2 =^ 2%) (28, 32, 38), postpartum hemorrhage (OR=0.63, 95%CI: 0.05-7.14, *P*=0.710, I^2 =^ 0%) (22), placental abruption (OR=1.23, 95%CI: 0.70-2.17, *P*=0.470, I^2 =^ 0%) (28, 32, 38), fetal growth restriction (OR=0.99, 95%CI: 0.72-1.37, *P*=0.970, I^2 =^ 0%) (32, 38), premature rupture of membranes (OR=0.80, 95%CI: 0.47-1.38, *P*=0.420, I^2 =^ 0%) (28) or low birth weight (OR=0.99, 95%CI: 0.72-1.37, *P*=0.970, I^2 =^ 0%) (22, 28, 32, 38) (**Table 3**).

TSA showed that the current evidences for preterm delivery and miscarriage were sufficient to reach firm conclusions, whereas the current evidences for gestational hypertension, gestational diabetes, postpartum hemorrhage, placental abruption, premature rupture of membranes, fetal growth restriction, fetal distress and low birth weight were insufficient and inconclusive (**Supplementary Figure 21–30**).

According to GRADE, the quality of evidence was rated as low for preterm delivery, miscarriage, and gestational hypertension; rated as very low for gestational diabetes, postpartum hemorrhage, placental abruption, premature rupture of membranes, fetal growth restriction, fetal distress, and low birth weight (**Supplementary Table 7**).

## 4 Discussion

### 4.1 Main findings

To our knowledge, this is the most comprehensive systematic review and meta-analysis assessing the effect of LT4 therapy in pregnant women with SCH and is the first study to investigate this effect using the TSA method. Our results showed that there were no statistically significant differences between LT4 group and control group in all outcomes. TSA showed that the results for all outcomes were insufficient and inconclusive. According to GRADE, the evidences for four outcomes (miscarriage, gestational hypertension, gestational diabetes, and small for gestational age) were rated as moderate quality, while the evidences for the other outcomes were rated as low or very low quality. However, in both the TPOAb-positive subgroup and the TPOAb-negative subgroup, LT4 therapy was associated with reduced risks of many outcomes, such as preterm delivery, miscarriage, gestational hypertension, gestational diabetes, et al. But the quality of evidence for these outcomes was low or very low.

### 4.2 Compared with previous studies

Our systematic review and meta-analysis found no evidence of benefit of LT4 therapy on pregnancy, neonatal and childhood outcomes in pregnant women with SCH, which was inconsistent with previous systematic reviews and meta-analyses adopting the old 2011 ATA diagnostic criteria. For example, the meta-analysis of Rao et al. (2019) (39) showed that LT4 therapy was associated with reduced risks of pregnancy loss and preterm birth, the meta-analysis of Nazarpour et al. (2019) (39, 40) showed that LT4 therapy was associated with reduced risk of pregnancy loss, and the meta-analysis of Bein et al. (2021) (39, 41) showed that LT4 therapy was associated with reduced risks of pregnancy loss and neonatal death. Currently, the new 2017 ATA diagnostic criteria is the most commonly accepted and widely used diagnostic standard in the clinic, which is quite different from the old 2011 ATA diagnostic criteria. Thus, these previous systematic reviews and meta-analyses were subject to misclassification bias and cannot reflect the real clinical situation.

Recently Ding et al. (8) published a systematic review and meta-analysis based on the new 2017 ATA diagnostic criteria, and found that LT4 therapy was associated with reduced risks of pregnancy loss, preterm delivery, and gestational hypertension. However, this study suffers from a series of problems, such as directly combining RCTs and cohort studies for meta-analysis, not searching some important literature databases, lacking some important outcomes, not performing TSA analysis, not evaluating the quality of evidence by using GRADE method, and so on. By resolving these problems, our systematic review and meta-analysis provided more comprehensive and reliable results than the systematic review and meta-analysis of Ding et al.

### 4.3 Explain unexpected findings

In our main meta-analysis, we found no statistically significant differences between LT4 group and control group in all primary and secondary outcomes. However, in both the TPOAb-positive subgroup and the TPOAb-negative subgroup, LT4 therapy was associated with reduced risks of many outcomes, such as preterm delivery, miscarriage, gestational hypertension, gestational diabetes, et al. The inconsistency between the results of our main meta-analysis and subgroup analysis was mainly due to the differences in quality and quantity of included studies. For each outcome, both the number of included studies and the number of studies with low and moderate risk of bias in the main meta-analysis were much more than those in the subgroup analysis. Moreover, according to GRADE, the evidences for four outcomes (miscarriage, gestational hypertension, gestational diabetes, and small for gestational age) in the main meta-analysis were rated as moderate quality, whereas the evidences for all outcomes in the subgroup analysis were rated down to low or very low quality. Thus, the main meta-analysis results are more credible than the subgroup analysis results, and our study are more supportive of the viewpoint that LT4 therapy has little benefit in pregnant women with SCH.

Another issue to note was that although our main meta-analysis of RCTs showed no statistically significant differences, there were trends toward decreased risks of some outcomes, such as preterm delivery, miscarriage, placental abruption and low birth weight. Moreover, TSA showed that the current RCTs for these outcomes did not have enough statistical power to reach firm conclusions. Thus, these negative results of our meta-analysis might be due to the relatively small sample size and be altered by future high quality and large sample size RCTs.

### 4.4 Clinical and research recommendations

Nowadays, LT4 has been widely used to treat SCH during pregnancy. Particularly in China, nearly all pregnant women with SCH receive LT4 therapy (32). Furthermore, the two most widely accepted guidelines, the 2017 ATA guideline and the 2019 CMA guideline, all recommend LT4 therapy for SCH during pregnancy, although the strength of recommendations differs by TPOAb status in the 2017 ATA guideline. However, our results suggest that LT4 therapy has no evidence of benefit in treatment of SCH during pregnancy. Thus, based on our results, the widespread use of LT4 in pregnant women with SCH and the recommendations of these two guidelines may not be appropriate. Moreover, the 2020 ACOG guideline does not recommend LT4 therapy for SCH during pregnancy, which is supported by our results and need to be taken into consideration in future clinical practice.

As the relatively small sample size of current studies limited the statistical power, further high quality and large sample size RCTs are still needed to reach a firm conclusion on the effect of LT4 therapy in pregnant women with SCH. In addition, the current evidences for both the TPOAb-positive subgroup and the TPOAb-negative subgroup are low or very low quality and cannot be used to guide clinical decision-making, future high quality RCTs also need to expand the focus to explore the different roles of LT4 in TPOAb-positive and TPOAb-negative women.

### 4.5 Strengths and limitations

Our systematic review and meta-analysis has several strengths. First, we adopted the new 2017 ATA diagnostic criteria for SCH during pregnancy, which could avoid misclassification bias compared with those systematic reviews and meta-analyses adopting the old 2011 ATA diagnostic criteria. Second, our study involved a comprehensive search of the literature, a broad range of clinical outcomes, and an in-depth discussion of the different roles of LT4 in TPOAb-positive and TPOAb-negative women, which ensured the comprehensiveness of our results. Third, our study used the most effective and reliable tools to evaluate the risk of bias and quality of evidence, and performed TSA to test whether the current evidence was sufficient, which ensured the reliability and accuracy of our results.

Our systematic review and meta-analysis also has some limitations. First, the number of included studies with low or moderate risk of bias on this topic was limited and the sample size of these studies was relatively small. This resulted in inadequate statistical power to draw firm conclusions for most outcomes. Second, the included studies differed in terms of LT4 dosage, with some studies using fixed dosages, while others titrated the dose to achieve a target TSH level. Third, we only included RCTs and cohort studies published in English or Chinese, which might lead to a language bias. Fourth, we cannot rule out the possibility of publication bias due to the relatively small number of included studies.

## 5 Conclusion

Unlike previous systematic reviews and meta-analyses, our study found no evidence of benefit of LT4 therapy on pregnancy, neonatal and childhood outcomes in pregnant women with SCH. These findings do not support LT4 therapy for SCH during pregnancy. However, although not statistically significant, there were trends toward decreased risk of some outcomes (such as preterm delivery or miscarriage), and the negative results for these outcomes might be due to the relatively small sample size. Thus, further high quality and large sample size RCTs are still needed to clarify this issue.

## Data availability statement

The original contributions presented in the study are included in the article/**Supplementary Material**. Further inquiries can be directed to the corresponding authors.

## Author contributions

LLZ, LZ, X-FJ and QW conceptualized the research question. X-FJ and MZ participated in drafting and writing the review. MZ, JC, X-FJ and LZ participated in the formulation of retrieval strategies, data acquisition, data analysis and quality assessment. DL, CZ, HL and KZ participated in the drawing of tables and figures. LLZ and LZ participated in critical revision of the manuscript. All authors contributed to the research and approved the final manuscript.

## Funding

This study was supported by Science and Technology Plan Project of Sichuan Province (2020YFS0035, 2019YFS0410). The funders had no role in the review design, conduct, interpretation, and writing of the report.

## Conflict of interest

The authors declare that the research was conducted in the absence of any commercial or financial relationships that could be construed as a potential conflict of interest.

## Publisher’s note

All claims expressed in this article are solely those of the authors and do not necessarily represent those of their affiliated organizations, or those of the publisher, the editors and the reviewers. Any product that may be evaluated in this article, or claim that may be made by its manufacturer, is not guaranteed or endorsed by the publisher.
