# Supplementary material for: The impact of levothyroxine therapy on the pregnancy, neonatal and childhood outcomes of subclinical hypothyroidism during pregnancy: An updated systematic review, meta-analysis and trial sequential analysis
Source: Front Endocrinol (Lausanne). 2022 Aug 5;13:964084. doi: 10.3389/fendo.2022.964084 (PMC9400061; doi:10.3389/fendo.2022.964084)
Supplement: Supplementary file 1 [file DataSheet_1.docx]

**List of Supplementary Figures and Tables**

**Supplementary Figure 1.** Risk of bias for each included RCT.

**Supplementary Figure 2-30.** The trial sequential analysis results.

**Supplementary Table 1.** Search strategy in PubMed.

**Supplementary Table 2.** Characteristics of the included studies.

**Supplementary Table 3.** Risk of bias for each included cohort study.

**Supplementary Table 4.** Meta-analysis results for secondary outcomes.

**Supplementary Table 5.** Grade evidence profile for each outcome.

**Supplementary Table 6.** Grade evidence profile for each outcome in the TPOAb-positive subgroup.

**Supplementary Table 7.** Grade evidence profile for each outcome in the TPOAb-negative subgroup.


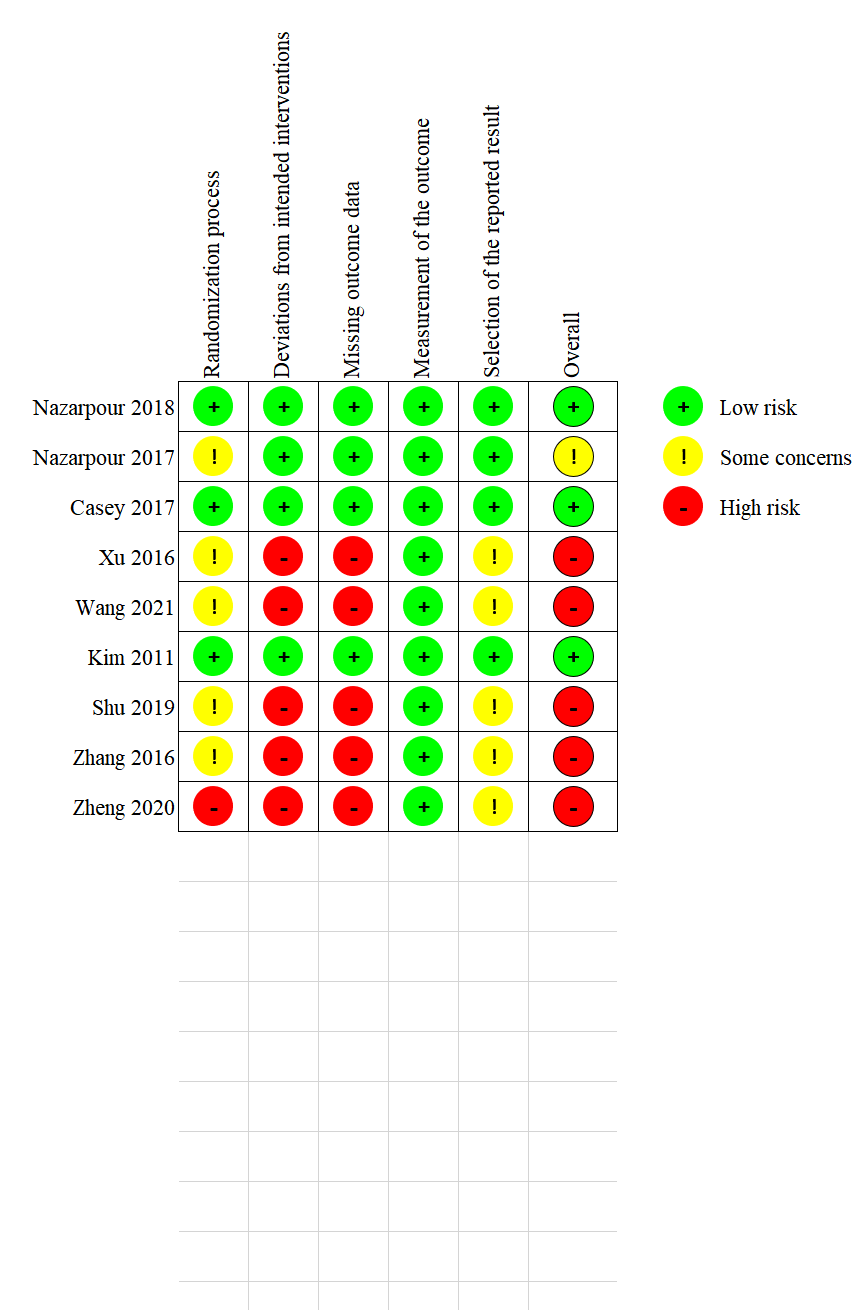


**Supplementary Figure 1.** Risk of bias for each included RCT.


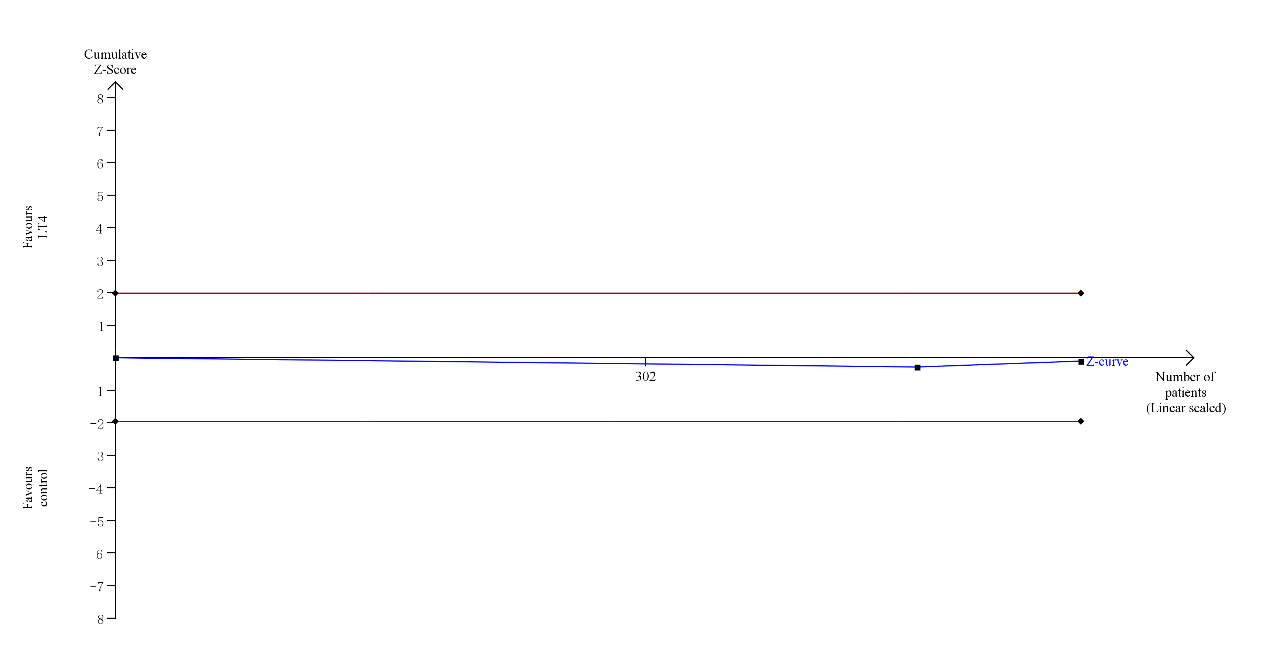


**Supplementary Figure 2.** Trial sequential analysis of postpartum hemorrhage. The risk of typeⅠerror was set at 5% with a power of 80%. The variance was calculated from the data obtained from the included trials. The relative risk reduction (RRR) was set at 20%.

**
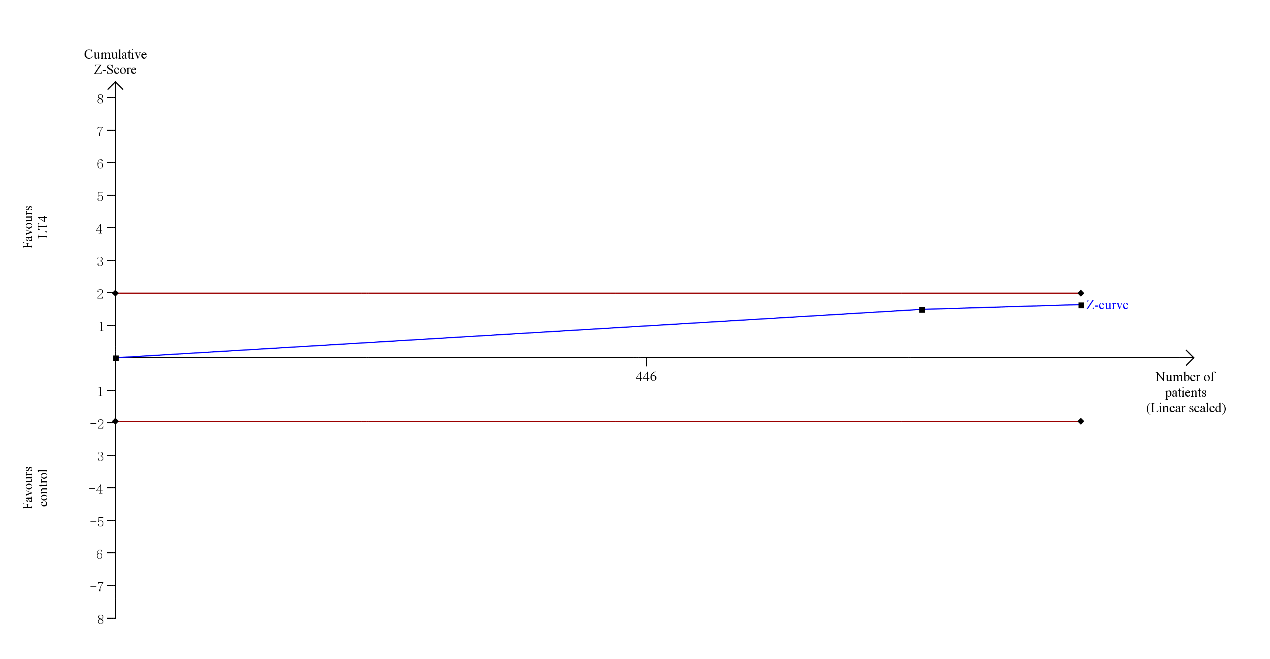
**

**Supplementary Figure 3.** Trial sequential analysis of placental abruption. The risk of typeⅠerror was set at 5% with a power of 80%. The variance was calculated from the data obtained from the included trials. The relative risk reduction (RRR) was set at 20%.


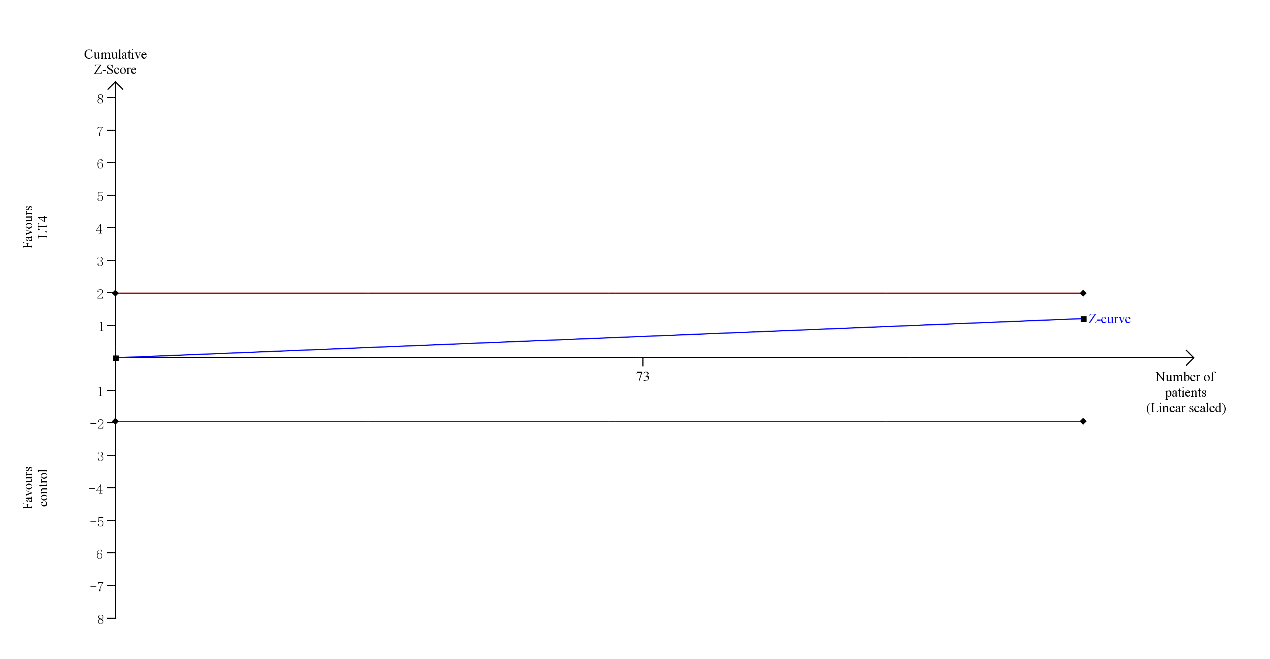


**Supplementary Figure 4.** Trial sequential analysis of fetal growth restriction. The risk of typeⅠerror was set at 5% with a power of 80%. The variance was calculated from the data obtained from the included trials. The relative risk reduction (RRR) was set at 20%.


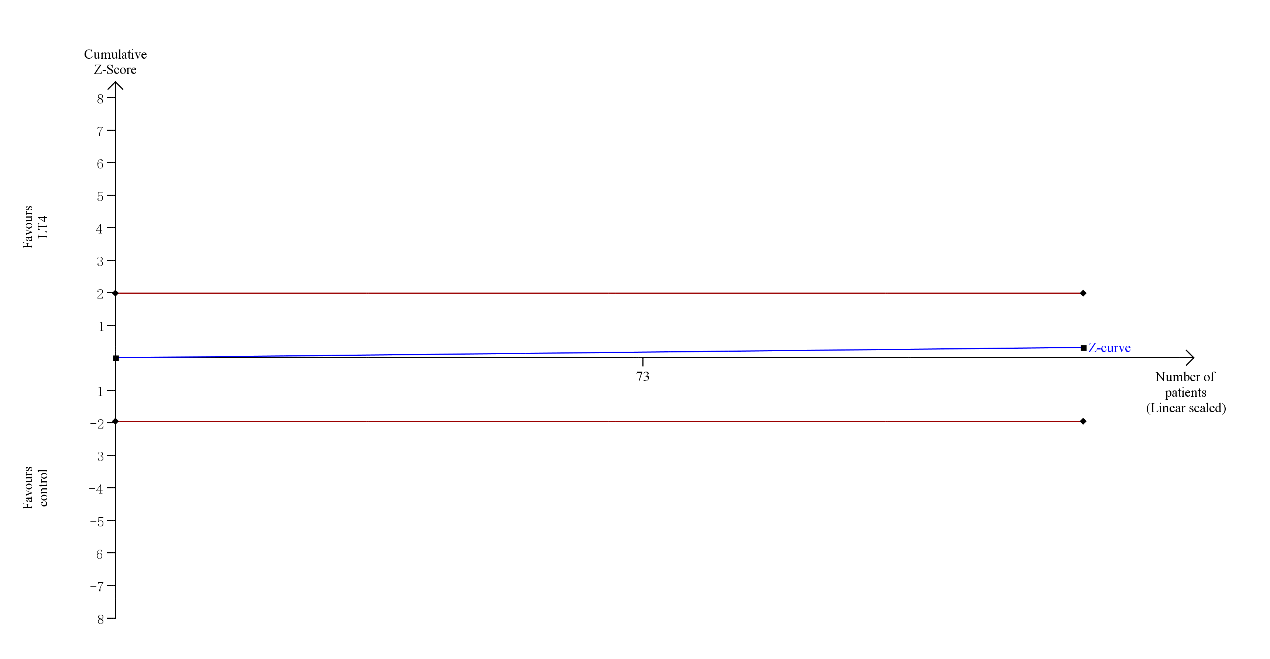


**Supplementary Figure 5.** Trial sequential analysis of fetal distress. The risk of typeⅠerror was set at 5% with a power of 80%. The variance was calculated from the data obtained from the included trials. The relative risk reduction (RRR) was set at 20%.


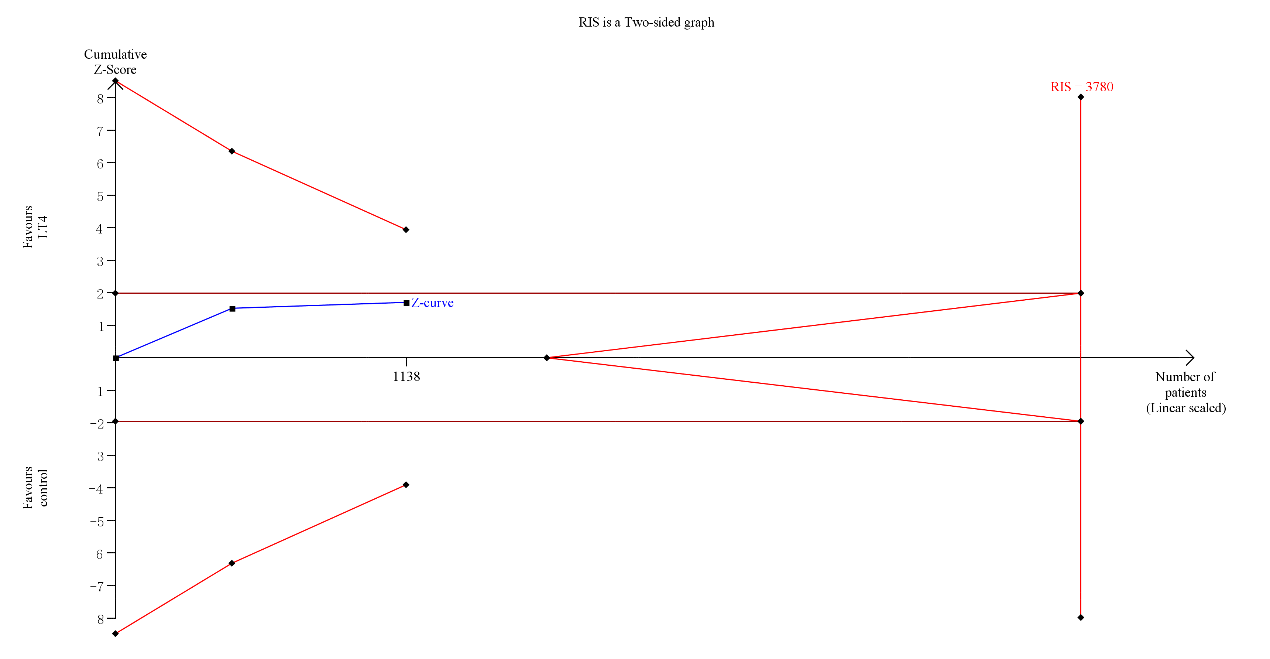


**Supplementary Figure 6.** Trial sequential analysis of premature rupture of membranes. The risk of typeⅠerror was set at 5% with a power of 80%. The variance was calculated from the data obtained from the included trials. The relative risk reduction (RRR) was set at 20%.


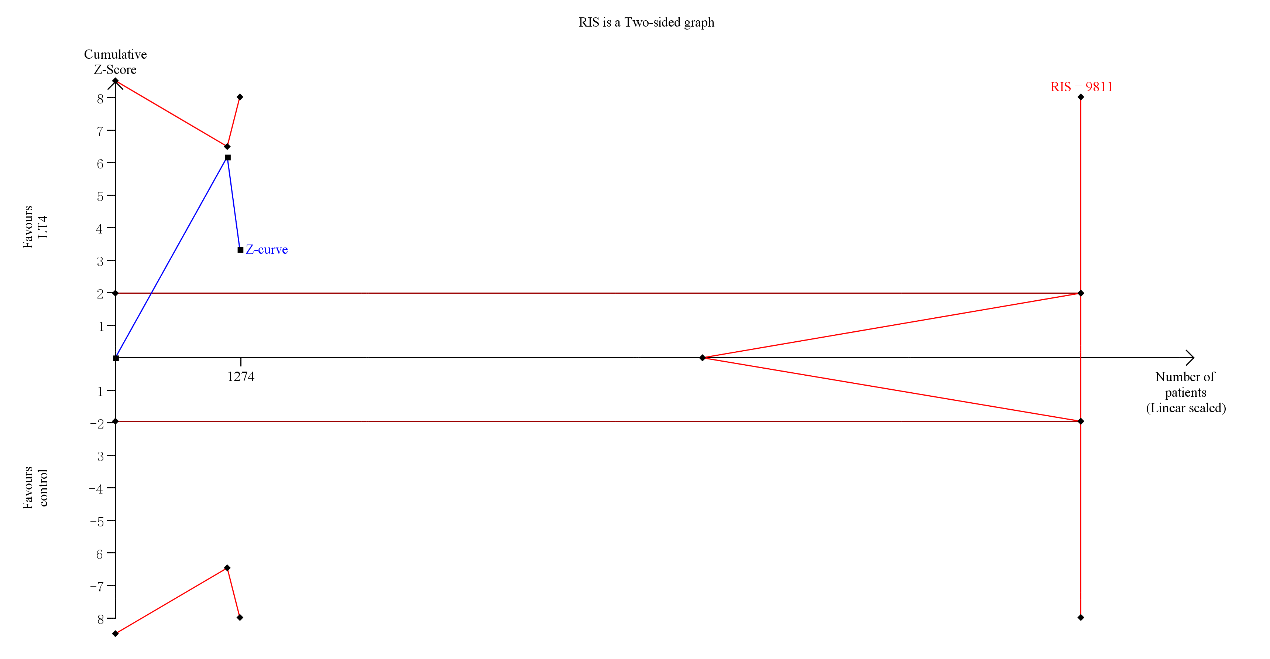


**Supplementary Figure 7.** Trial sequential analysis of low birth weight. The risk of typeⅠerror was set at 5% with a power of 80%. The variance was calculated from the data obtained from the included trials. The relative risk reduction (RRR) was set at 20%.


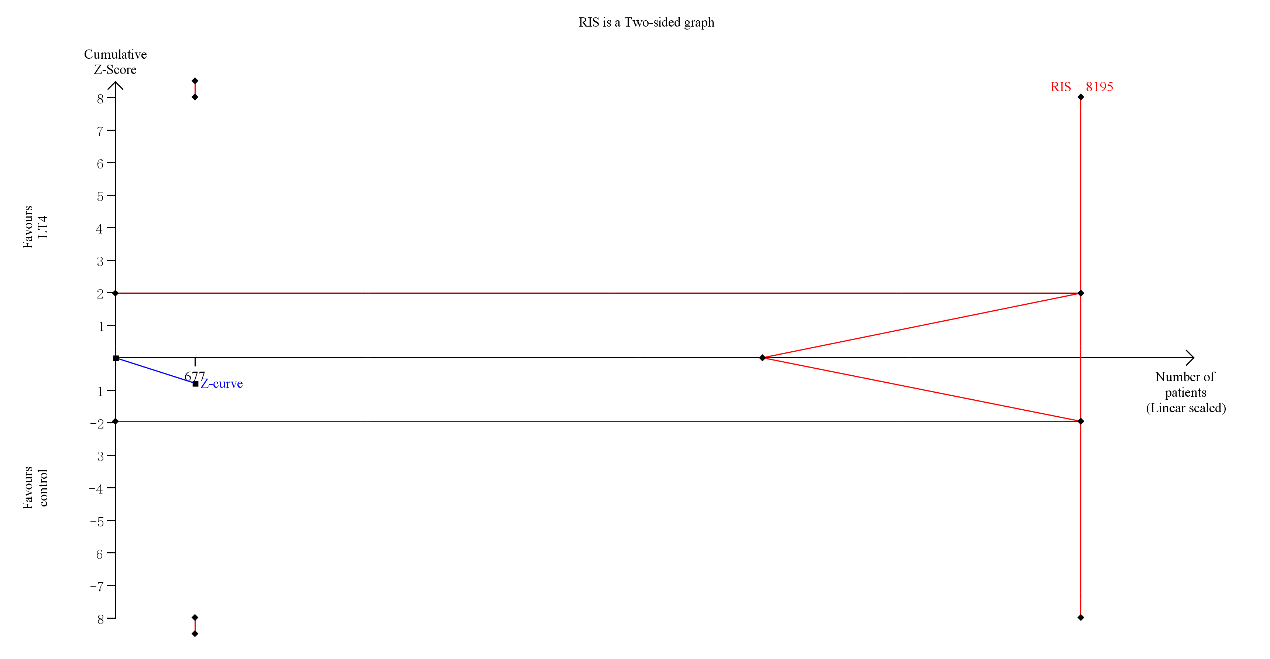


**Supplementary Figure 8.** Trial sequential analysis of small for gestational age. The risk of typeⅠerror was set at 5% with a power of 80%. The variance was calculated from the data obtained from the included trials. The relative risk reduction (RRR) was set at 20%.


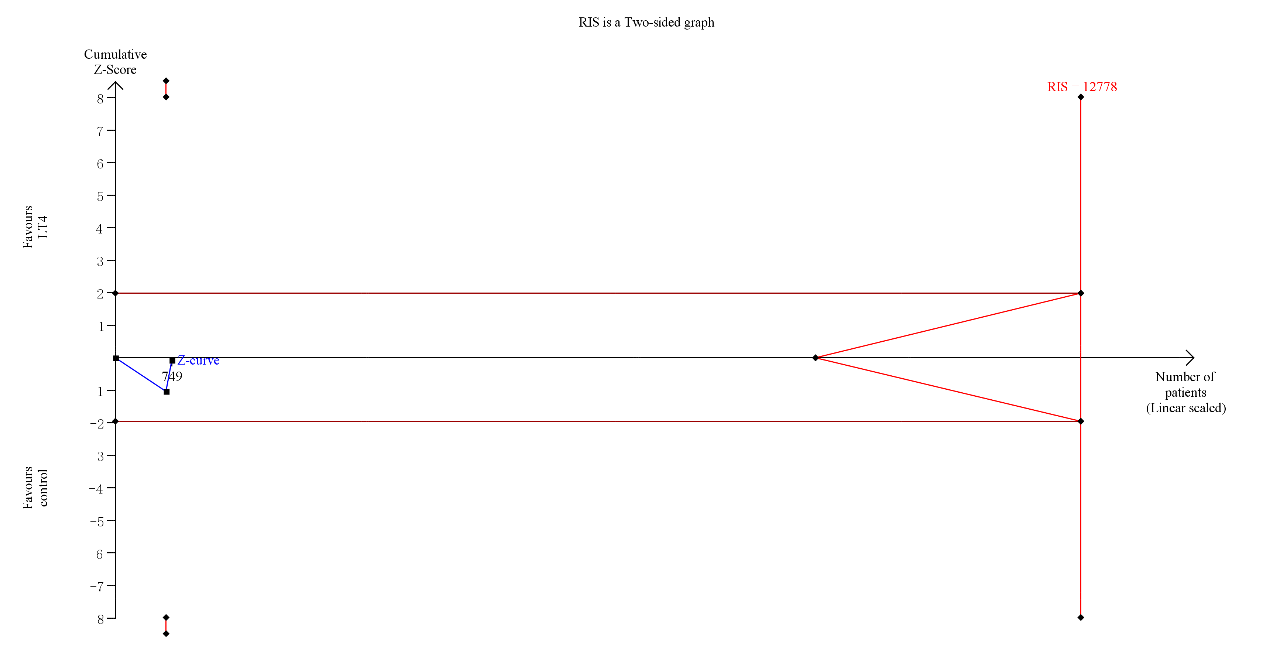


**Supplementary Figure 9.** Trial sequential analysis of neonatal intensive care unit (NICU) admission. The risk of typeⅠerror was set at 5% with a power of 80%. The variance was calculated from the data obtained from the included trials. The relative risk reduction (RRR) was set at 20%.


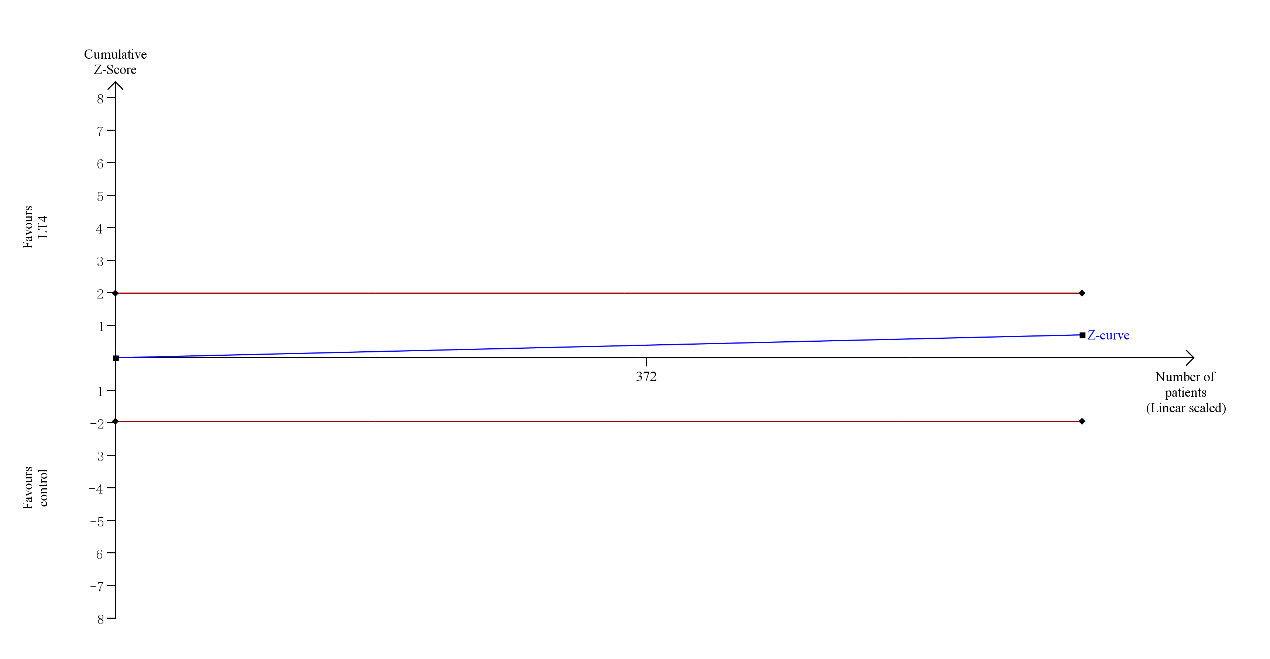


**Supplementary Figure 10.** Trial sequential analysis of neonatal death. The risk of typeⅠerror was set at 5% with a power of 80%. The variance was calculated from the data obtained from the included trials. The relative risk reduction (RRR) was set at 20%.


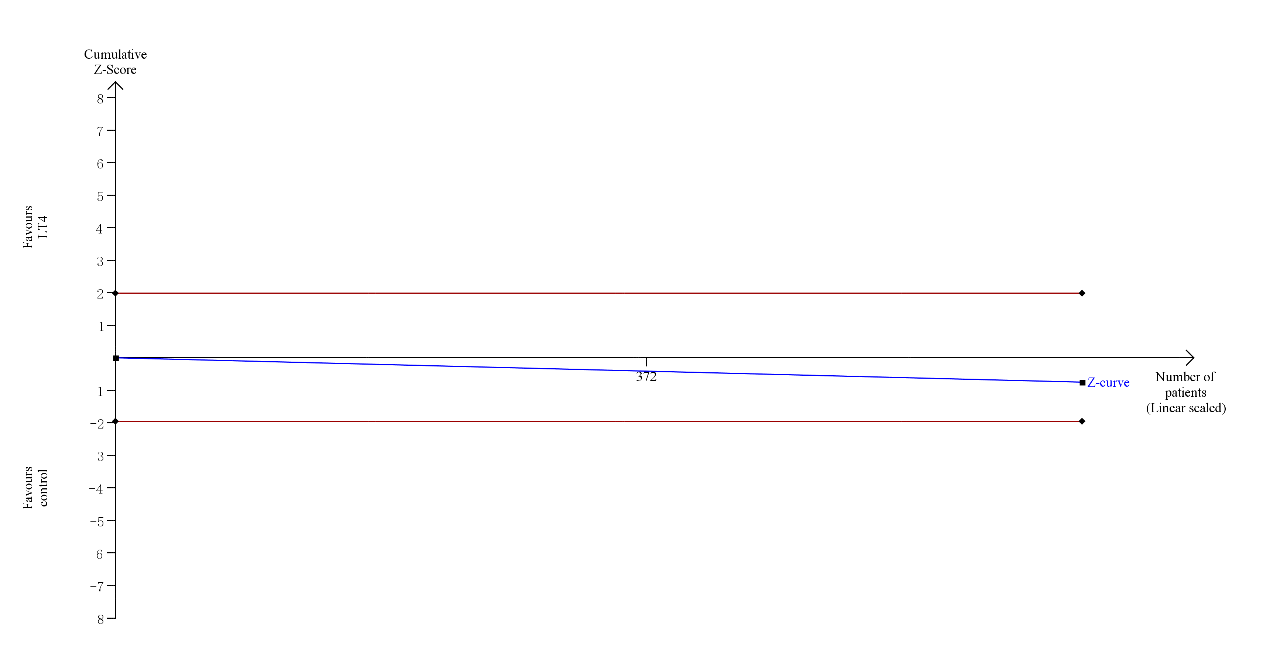


**Supplementary Figure 11.** Trial sequential analysis of respiratory distress syndrome. The risk of typeⅠerror was set at 5% with a power of 80%. The variance was calculated from the data obtained from the included trials. The relative risk reduction (RRR) was set at 20%.


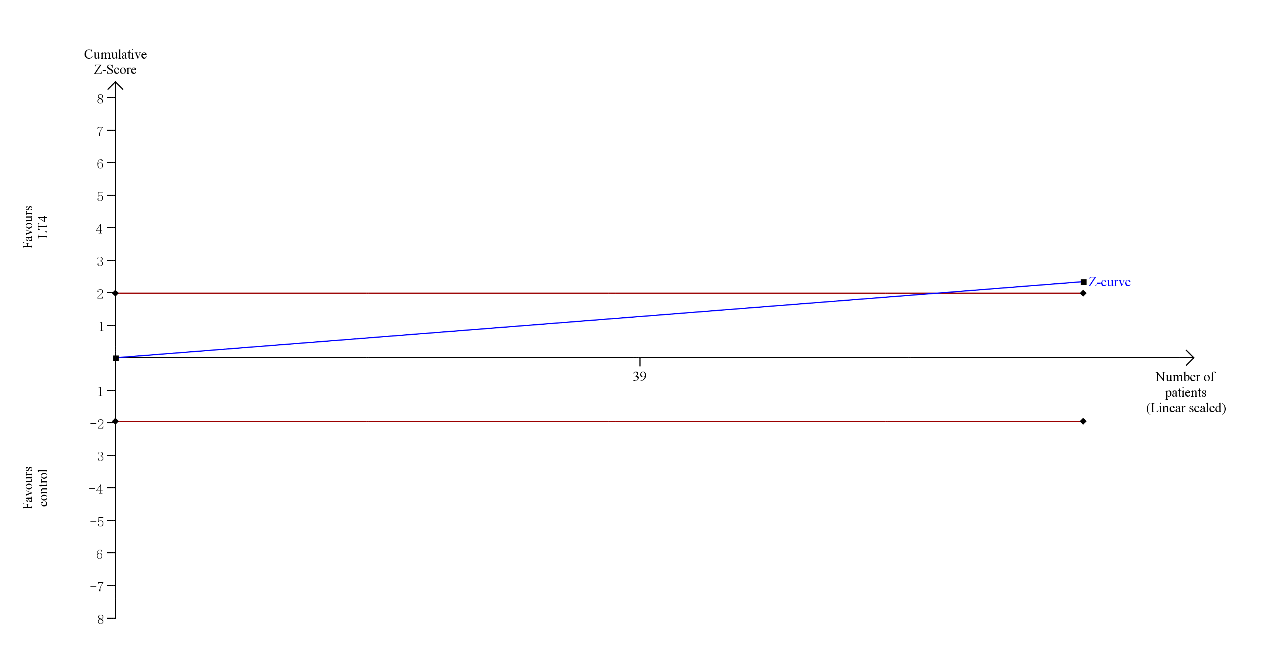


**Supplementary Figure 12.** Trial sequential analysis of preterm delivery in the TPOAb-positive subgroup. The risk of typeⅠerror was set at 5% with a power of 80%. The variance was calculated from the data obtained from the included trials. The relative risk reduction (RRR) was set at 20%.


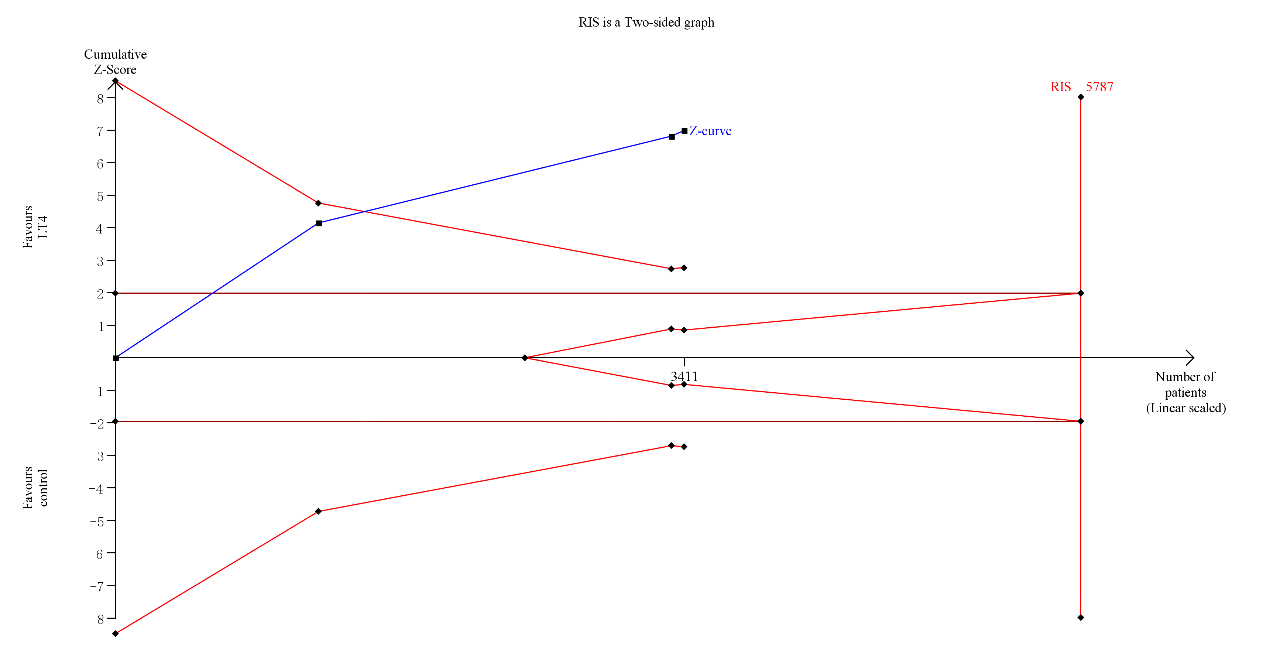


**Supplementary Figure 13.** Trial sequential analysis of miscarriage in the TPOAb-positive subgroup. The risk of typeⅠerror was set at 5% with a power of 80%. The variance was calculated from the data obtained from the included trials. The relative risk reduction (RRR) was set at 20%.


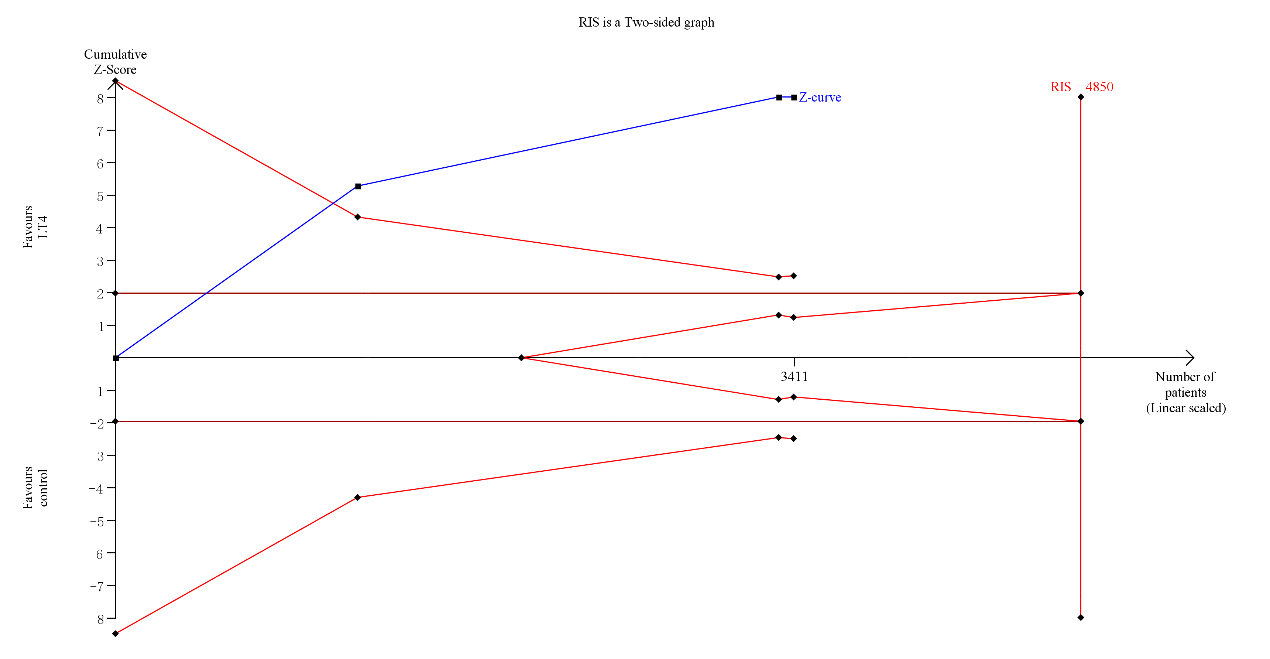


**Supplementary Figure 14.** Trial sequential analysis of gestational hypertension in the TPOAb-positive subgroup. The risk of typeⅠerror was set at 5% with a power of 80%. The variance was calculated from the data obtained from the included trials. The relative risk reduction (RRR) was set at 20%.


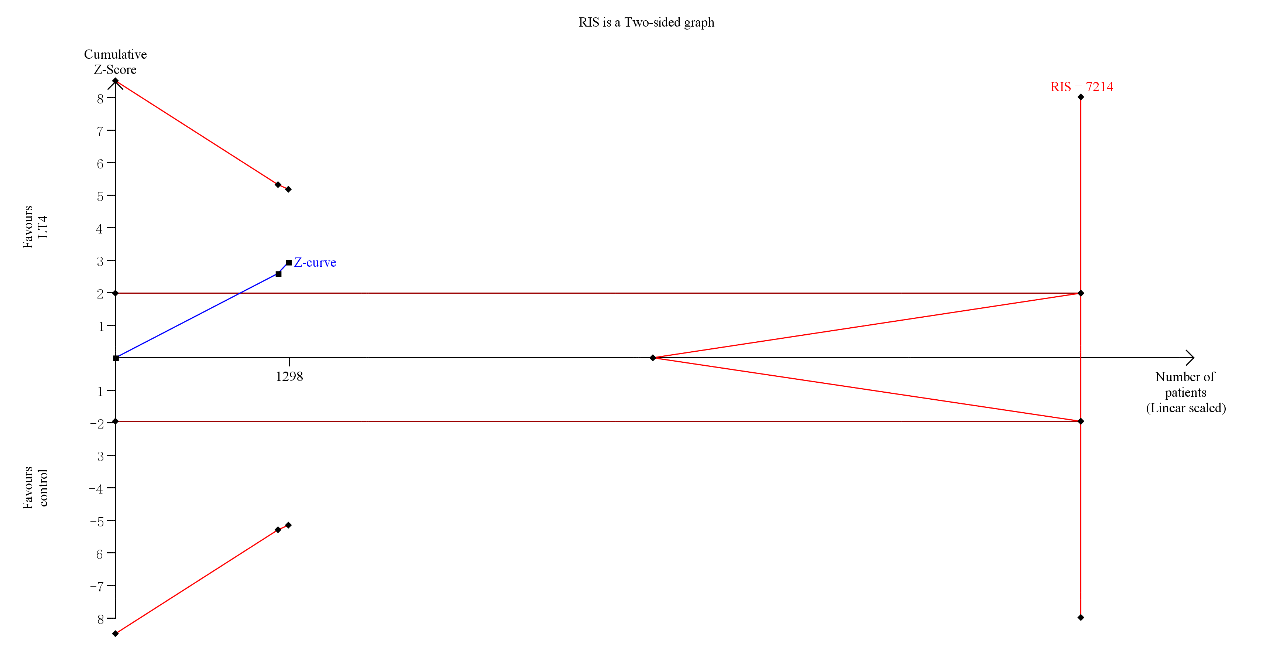


**Supplementary Figure 15.** Trial sequential analysis of gestational diabetes in the TPOAb-positive subgroup. The risk of typeⅠerror was set at 5% with a power of 80%. The variance was calculated from the data obtained from the included trials. The relative risk reduction (RRR) was set at 20%.


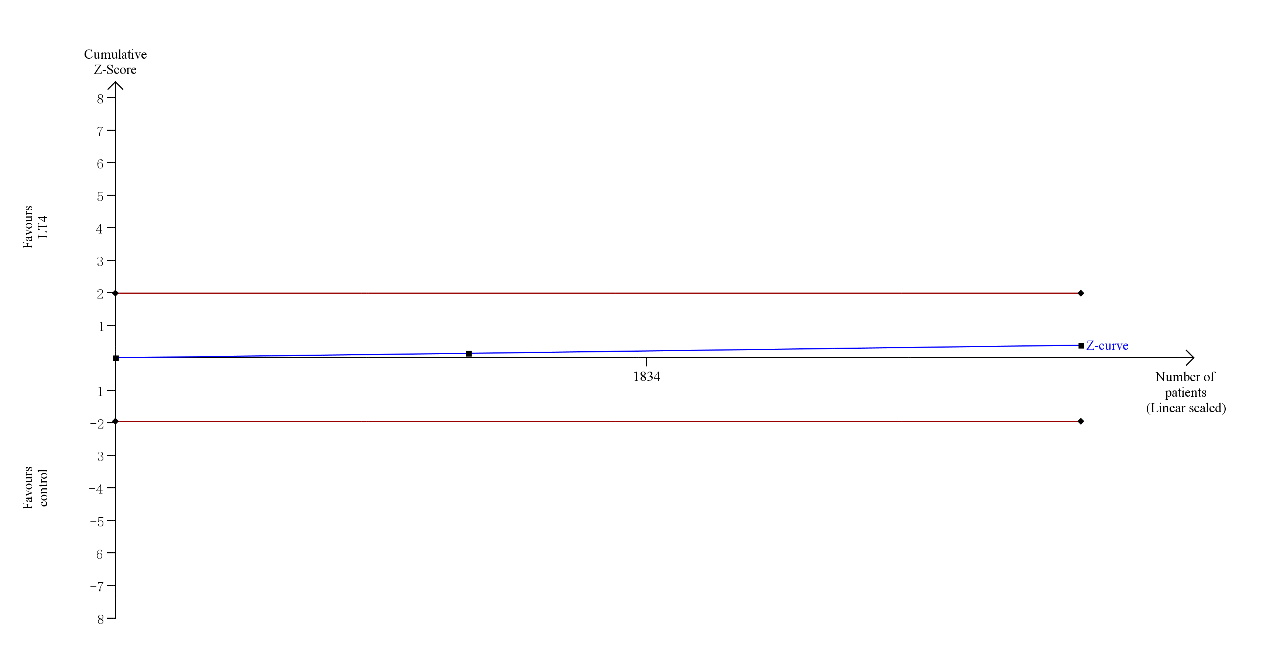


**Supplementary Figure 16.** Trial sequential analysis of placental abruption in the TPOAb-positive subgroup. The risk of typeⅠerror was set at 5% with a power of 80%. The variance was calculated from the data obtained from the included trials. The relative risk reduction (RRR) was set at 20%.


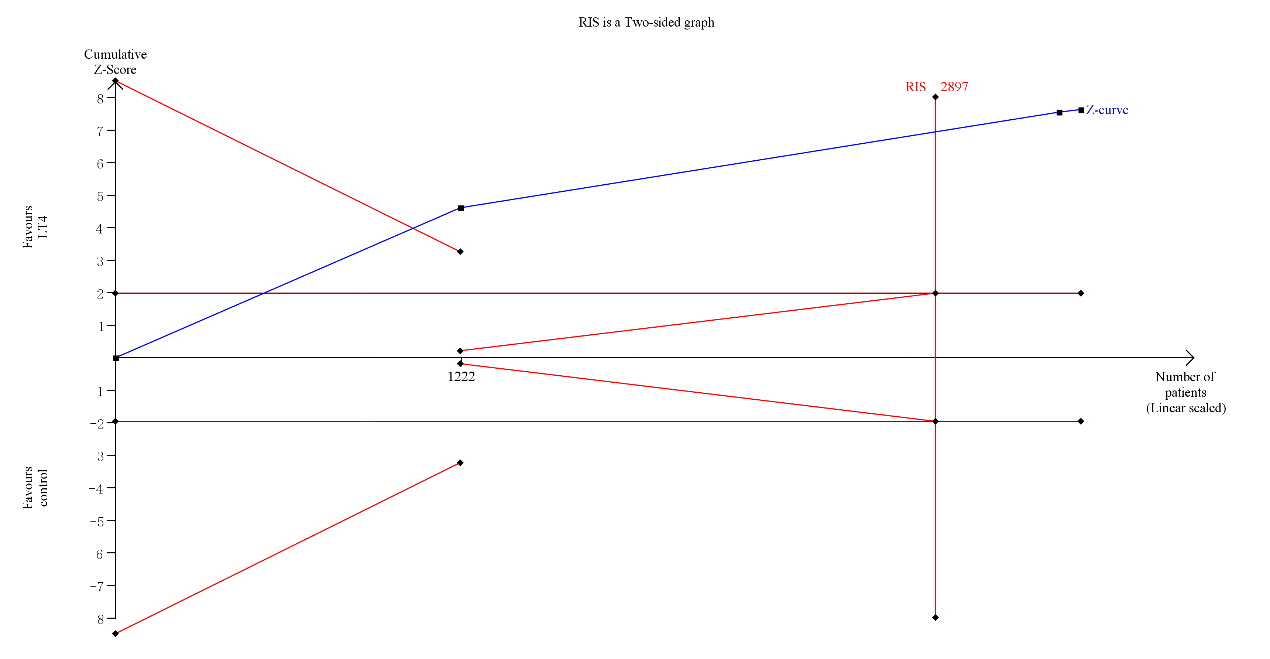


**Supplementary Figure 17.** Trial sequential analysis of fetal growth restriction in the TPOAb-positive subgroup. The risk of typeⅠerror was set at 5% with a power of 80%. The variance was calculated from the data obtained from the included trials. The relative risk reduction (RRR) was set at 20%.


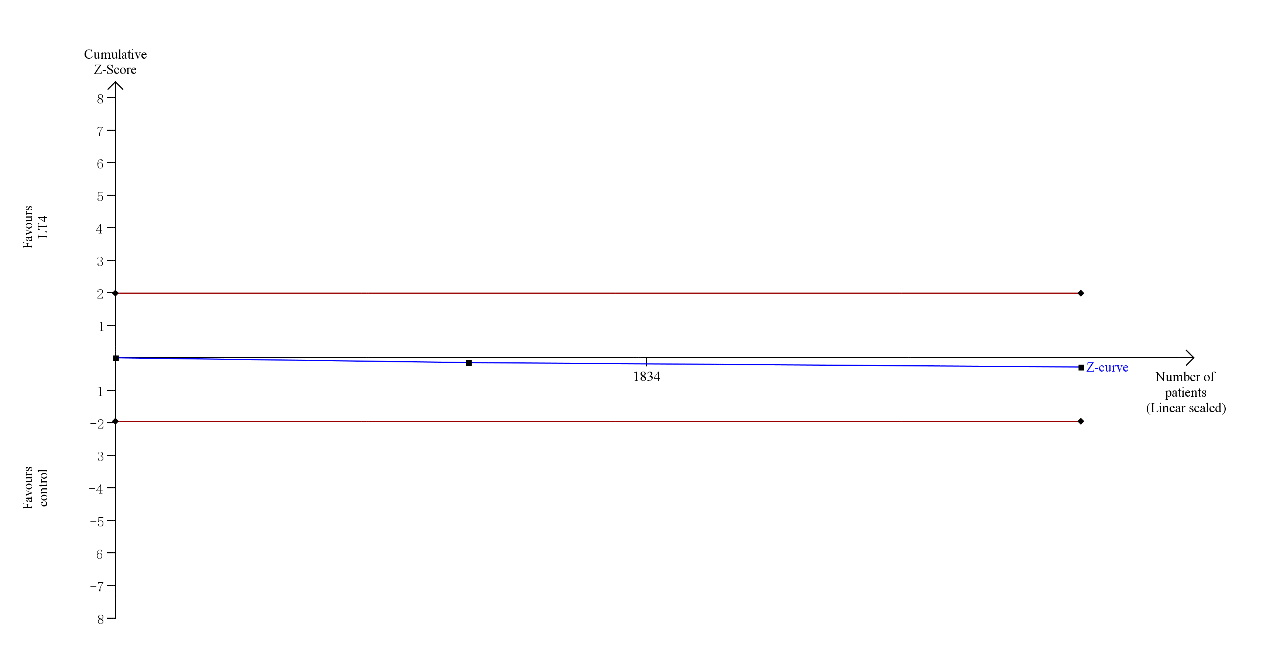


**Supplementary Figure 18.** Trial sequential analysis of fetal distress in the TPOAb-positive subgroup. The risk of typeⅠerror was set at 5% with a power of 80%. The variance was calculated from the data obtained from the included trials. The relative risk reduction (RRR) was set at 20%.


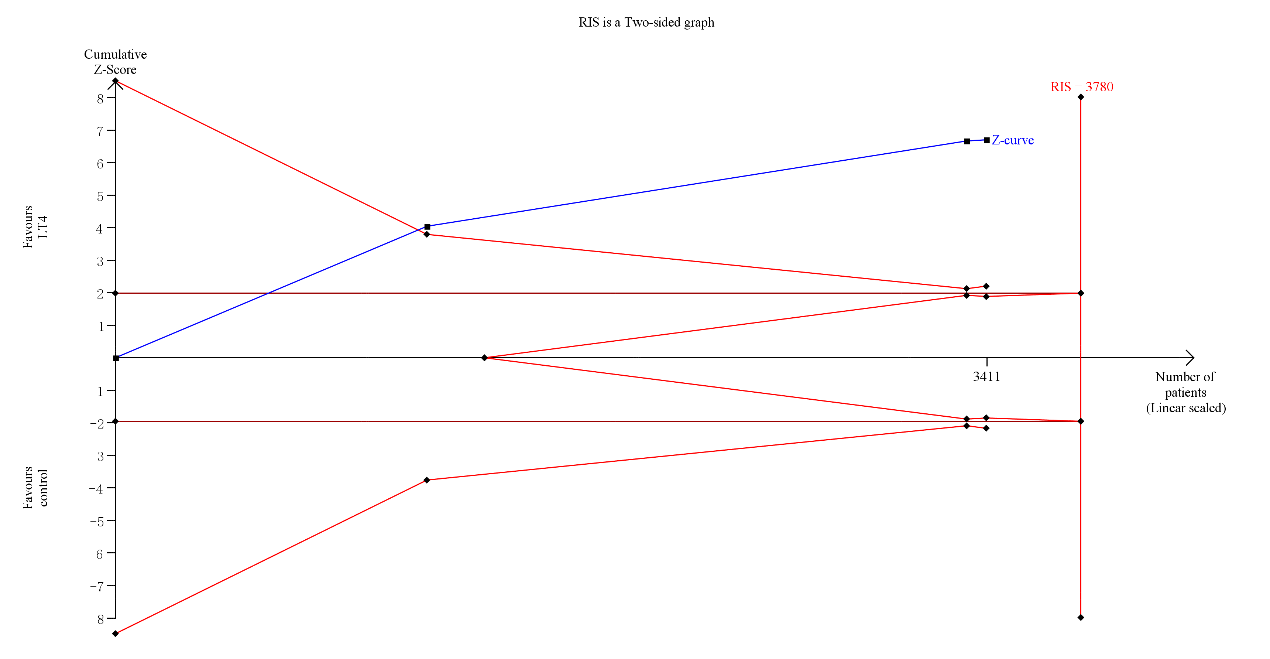


**Supplementary Figure 19.** Trial sequential analysis of low birth weight in the TPOAb-positive subgroup. The risk of typeⅠerror was set at 5% with a power of 80%. The variance was calculated from the data obtained from the included trials. The relative risk reduction (RRR) was set at 20%.


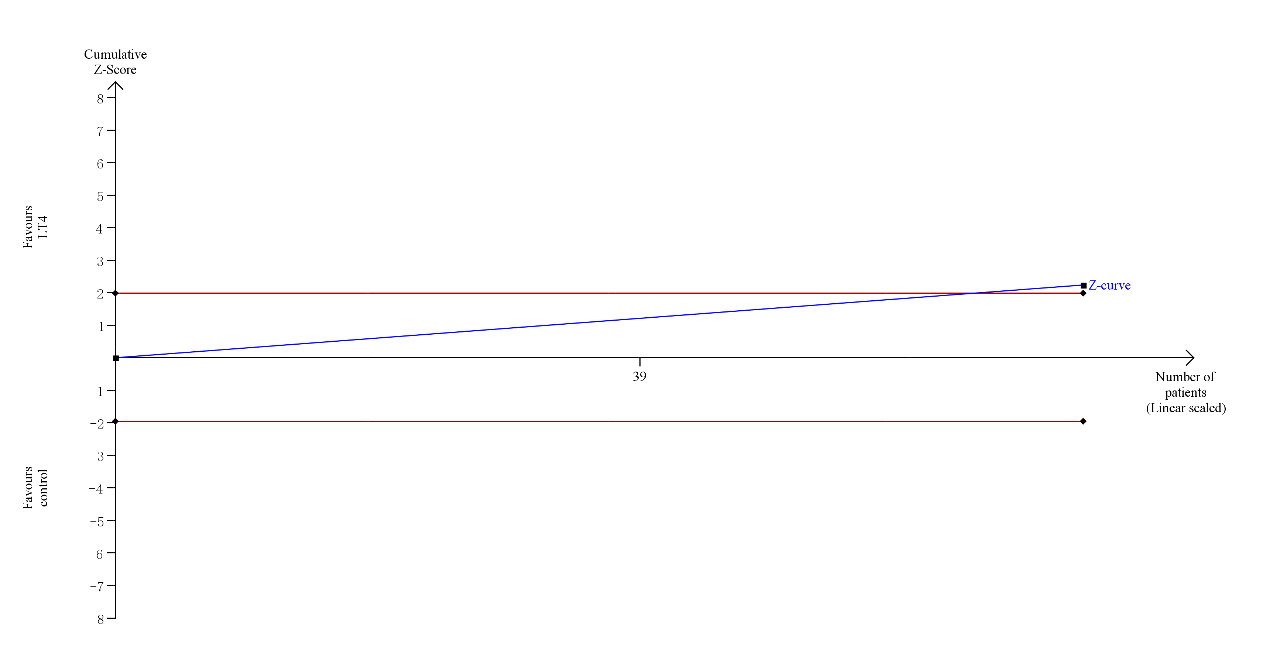


**Supplementary Figure 20.** Trial sequential analysis of neonatal intensive care unit (NICU) admission in the TPOAb-positive subgroup. The risk of typeⅠerror was set at 5% with a power of 80%. The variance was calculated from the data obtained from the included trials. The relative risk reduction (RRR) was set at 20%.


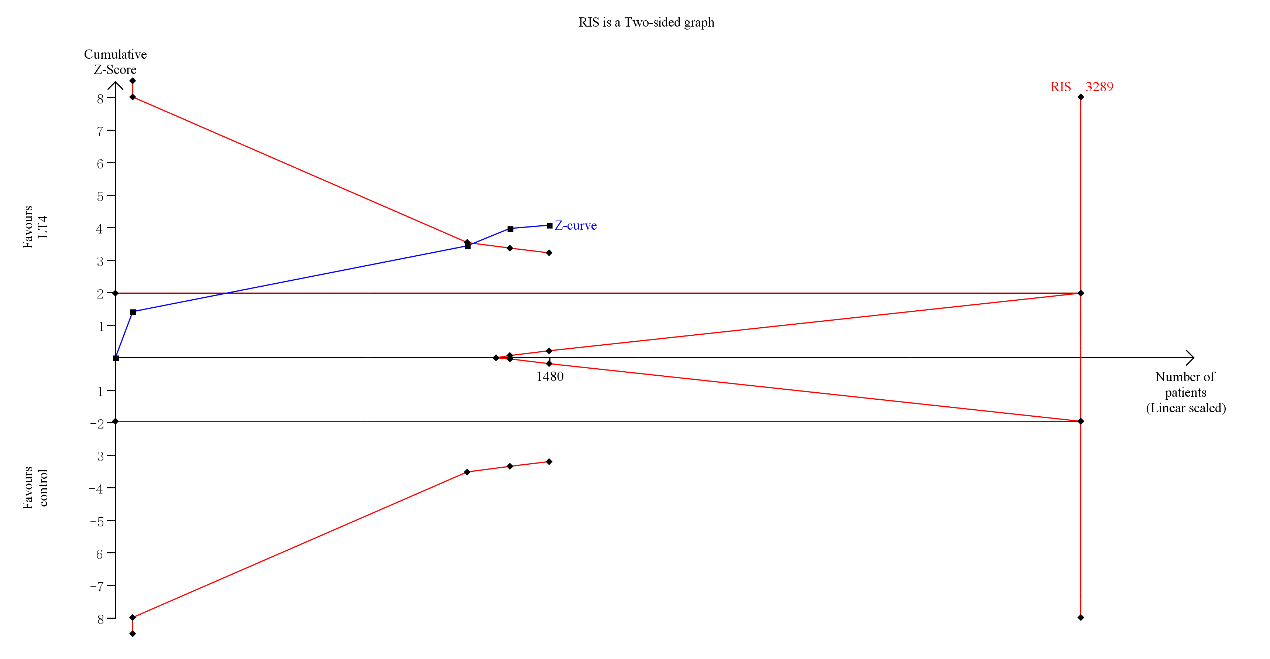


**Supplementary Figure 21.** Trial sequential analysis of preterm delivery in the TPOAb-negative subgroup. The risk of typeⅠerror was set at 5% with a power of 80%. The variance was calculated from the data obtained from the included trials. The relative risk reduction (RRR) was set at 20%.


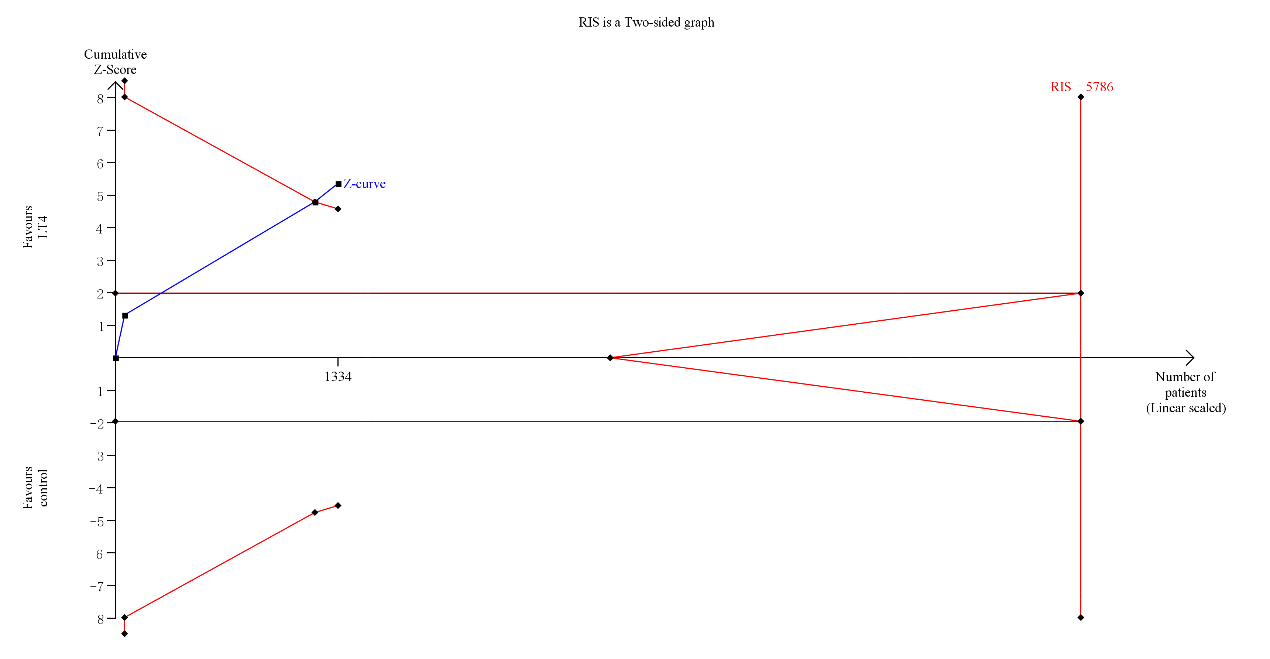


**Supplementary Figure 22.** Trial sequential analysis of miscarriage in the TPOAb-negative subgroup. The risk of typeⅠerror was set at 5% with a power of 80%. The variance was calculated from the data obtained from the included trials. The relative risk reduction (RRR) was set at 20%.


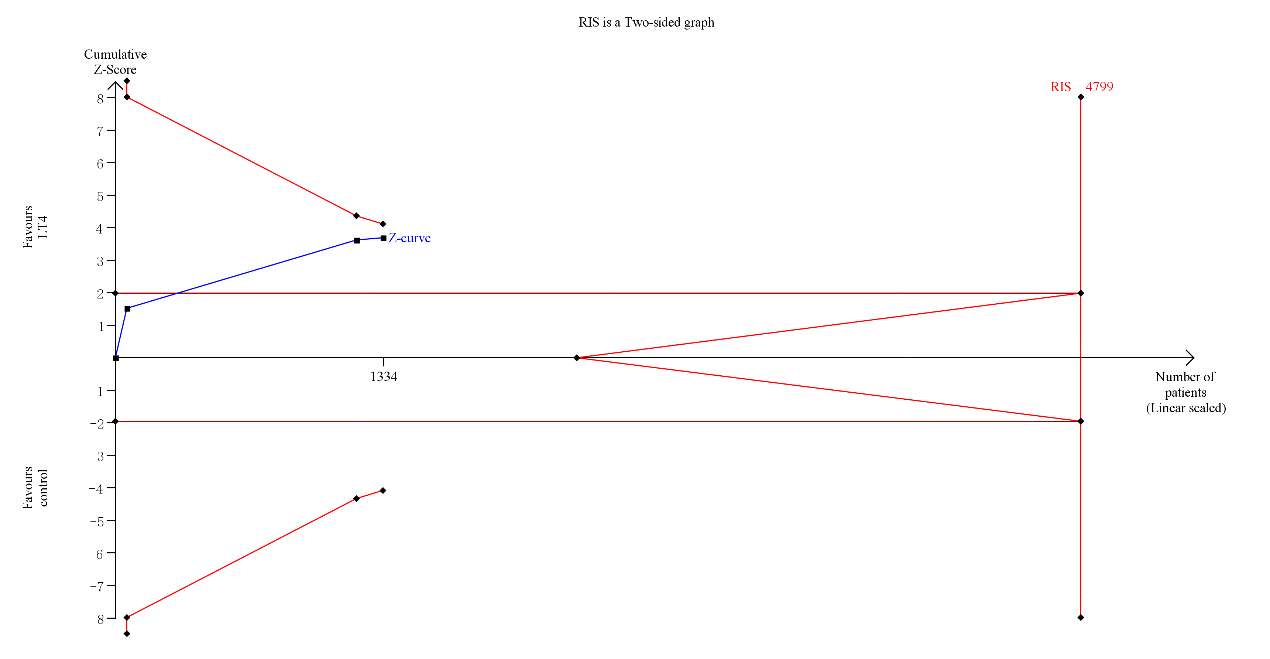


**Supplementary Figure 23.** Trial sequential analysis of gestational hypertension in the TPOAb-negative subgroup. The risk of typeⅠerror was set at 5% with a power of 80%. The variance was calculated from the data obtained from the included trials. The relative risk reduction (RRR) was set at 20%.


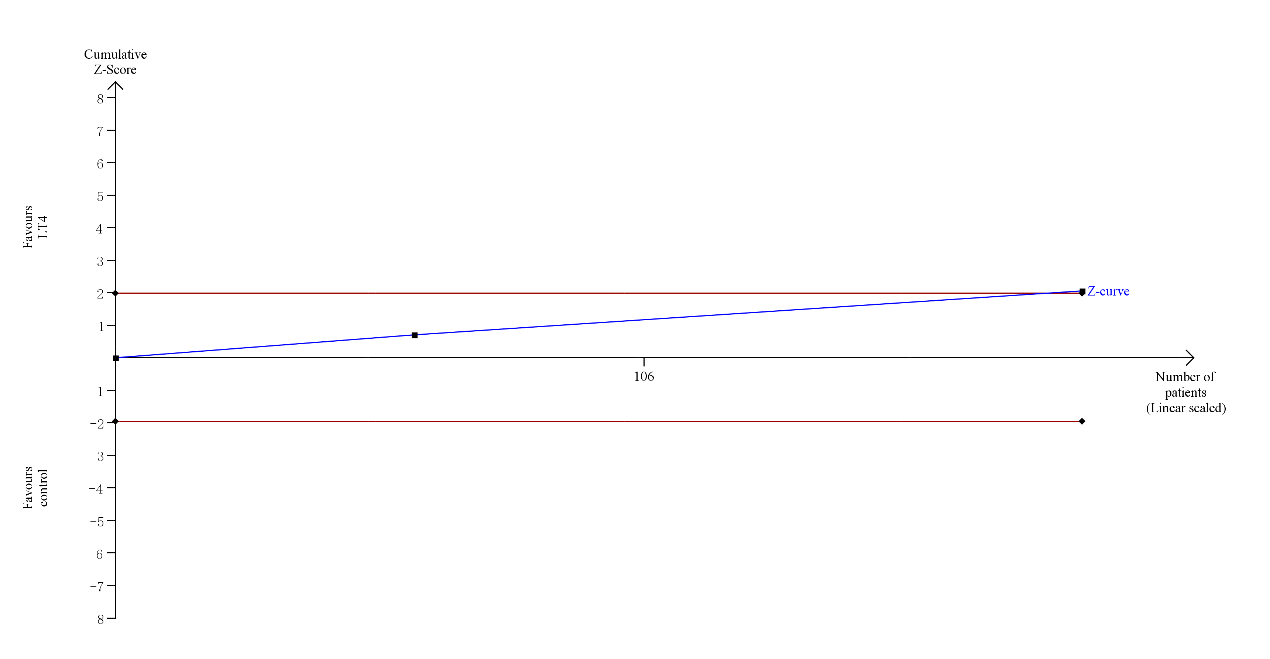


**Supplementary Figure 24.** Trial sequential analysis of gestational diabetes in the TPOAb-negative subgroup. The risk of typeⅠerror was set at 5% with a power of 80%. The variance was calculated from the data obtained from the included trials. The relative risk reduction (RRR) was set at 20%.


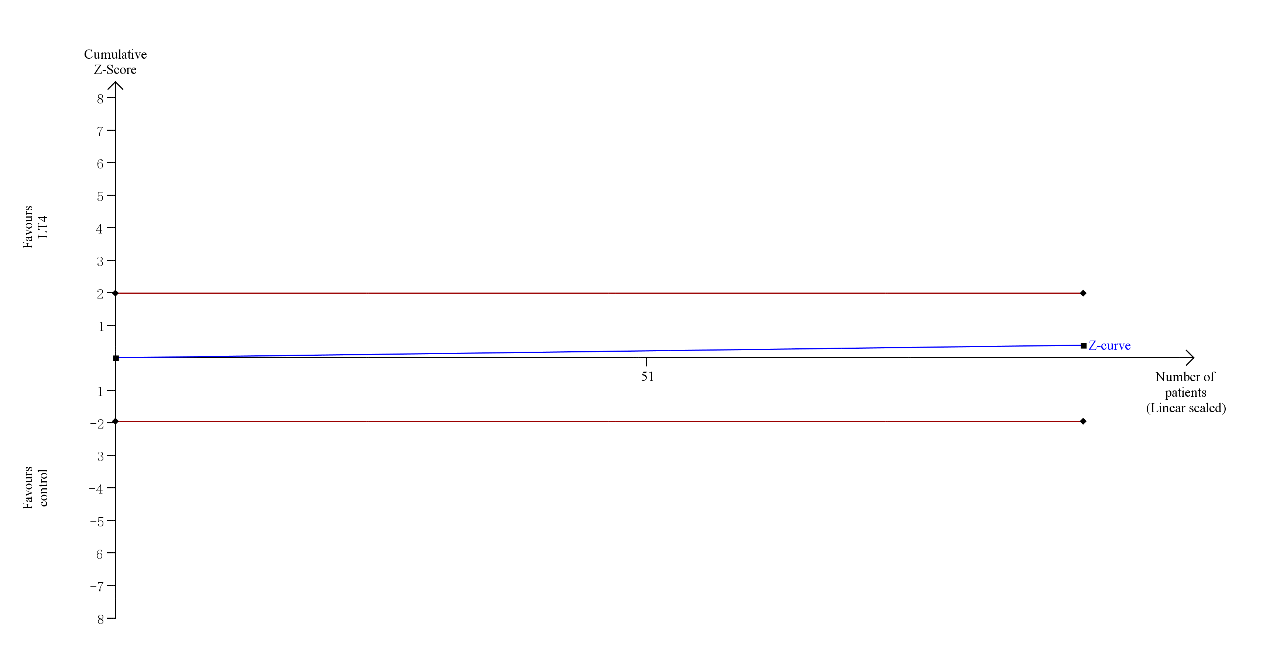


**Supplementary Figure 25.** Trial sequential analysis of postpartum hemorrhage in the TPOAb-negative subgroup. The risk of typeⅠerror was set at 5% with a power of 80%. The variance was calculated from the data obtained from the included trials. The relative risk reduction (RRR) was set at 20%.


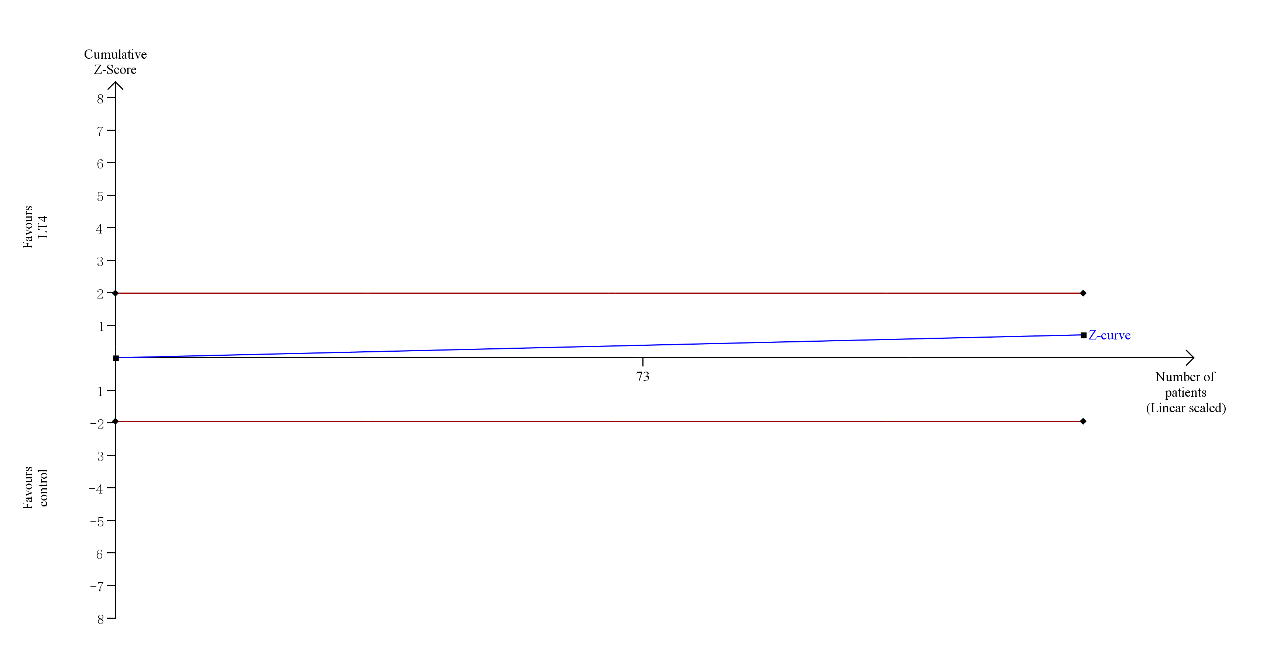


**Supplementary Figure 26.** Trial sequential analysis of placental abruption in the TPOAb-negative subgroup. The risk of typeⅠerror was set at 5% with a power of 80%. The variance was calculated from the data obtained from the included trials. The relative risk reduction (RRR) was set at 20%.


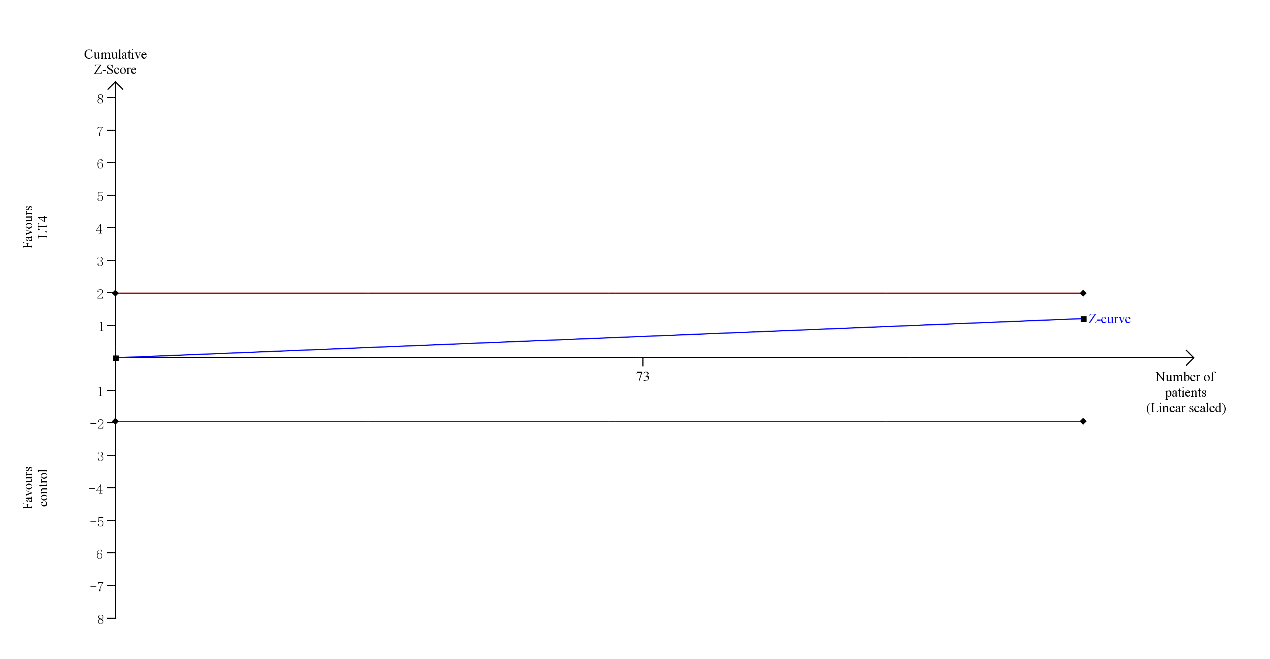


**Supplementary Figure 27.** Trial sequential analysis of fetal growth restriction in the TPOAb-negative subgroup. The risk of typeⅠerror was set at 5% with a power of 80%. The variance was calculated from the data obtained from the included trials. The relative risk reduction (RRR) was set at 20%.


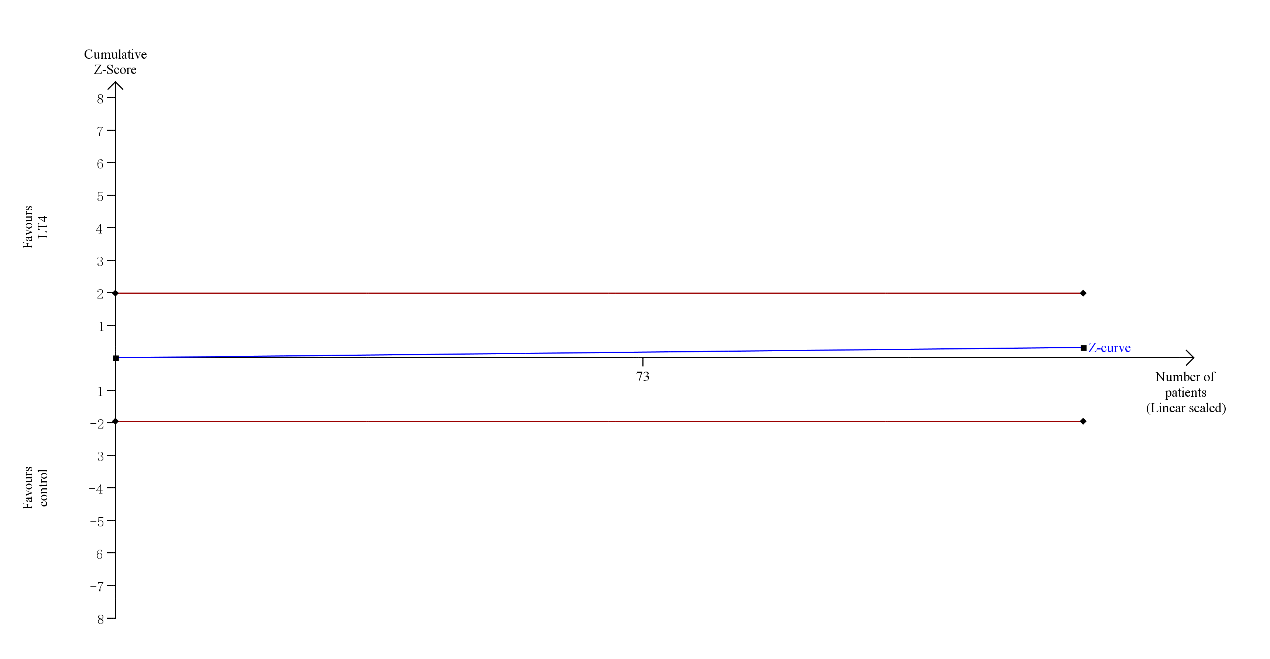


**Supplementary Figure 28.** Trial sequential analysis of fetal distress in the TPOAb-negative subgroup. The risk of typeⅠerror was set at 5% with a power of 80%. The variance was calculated from the data obtained from the included trials. The relative risk reduction (RRR) was set at 20%.


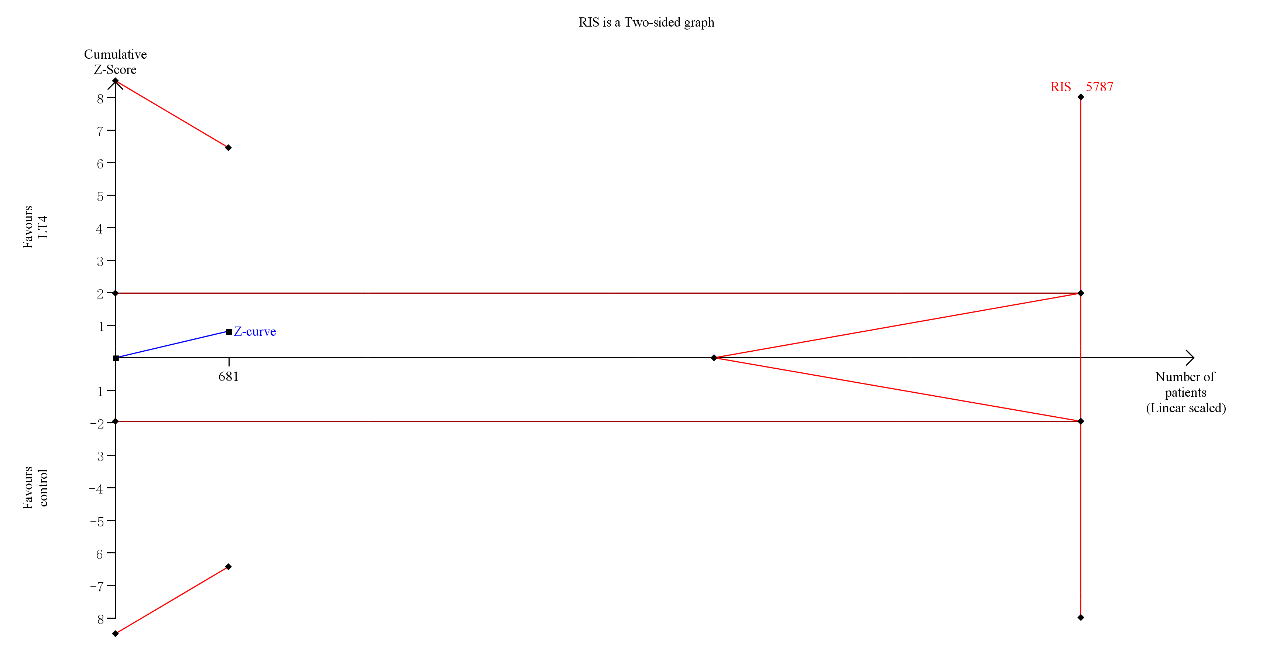


**Supplementary Figure 29.** Trial sequential analysis of premature rupture of membranes in the TPOAb-negative subgroup. The risk of typeⅠerror was set at 5% with a power of 80%. The variance was calculated from the data obtained from the included trials. The relative risk reduction (RRR) was set at 20%.


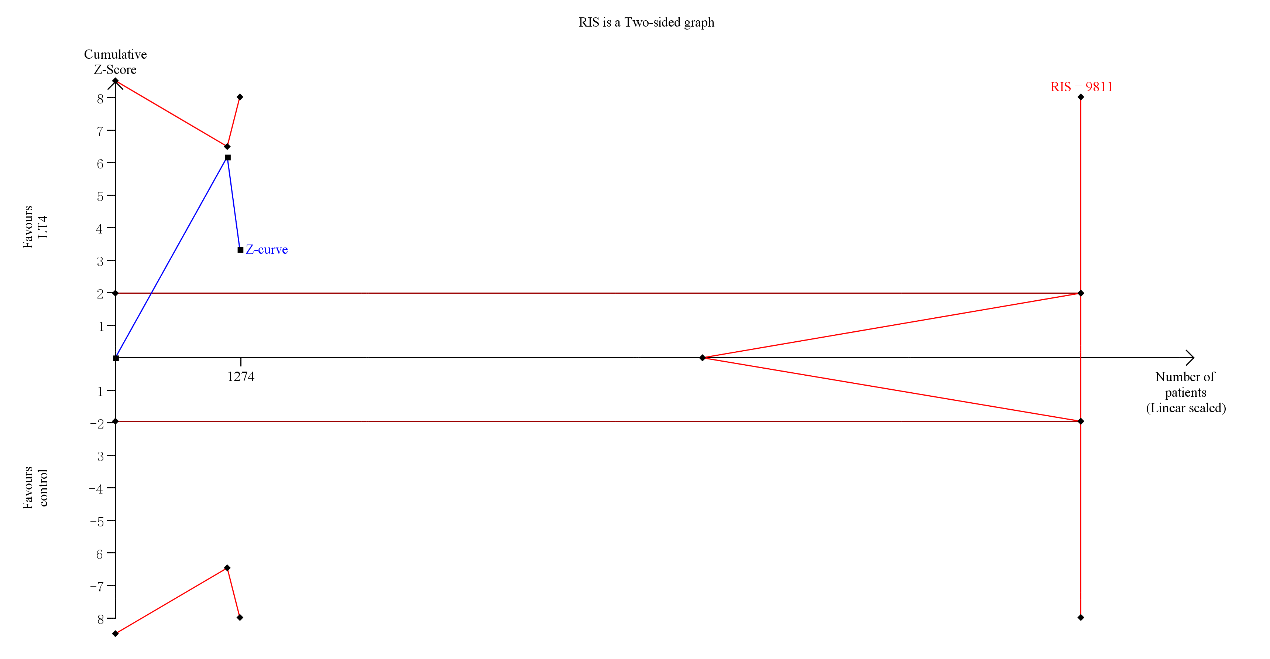


**Supplementary Figure 30.** Trial sequential analysis of low birth weight in the TPOAb-negative subgroup. The risk of typeⅠerror was set at 5% with a power of 80%. The variance was calculated from the data obtained from the included trials. The relative risk reduction (RRR) was set at 20%.

**Supplementary Table 1.** Search strategy in PubMed.

PubMed 02. 2022

| #1 | "subclinical"[Title/Abstract] OR "sub-clinical"[Title/Abstract] | 49901 |
| --- | --- | --- |
| #2 | "hypothyroidism"[MeSH Terms] OR "hypothyr*"[Title/Abstract] OR "hypo thyr*"[Title/Abstract] OR "thyroid deficien*"[Title/Abstract] OR "thyroid insufficien*"[Title/Abstract] | 51197 |
| #3 | "pregnancy"[MeSH Terms] OR "pregnanc*"[Title/Abstract] OR "pregnant"[Title/Abstract] OR "gestat*"[Title/Abstract] | 1140906 |
| #4 | "thyroxine"[MeSH Terms] OR "levothyroxine"[Title/Abstract] OR "LT4"[Title/Abstract] OR "thyroxine supplementation"[Title/Abstract] OR "thyroxine"[Title/Abstract] OR "synthroid"[Title/Abstract] | 62241 |
| #5 | #1 AND #2 AND #3 AND #4 | 466 |

**Supplementary Table 2.** Characteristics of the included studies.

| **Study** | **Country** | **Study design** | **Sample size** | | **Age (year)** | | **BMI (kg/m2)** | | **TSH level** | **Outcomes** |
| --- | --- | --- | --- | --- | --- | --- | --- | --- | --- | --- |
|  |  |  | **LT4** | **Control** | **LT4** | **Control** | **LT4** | **Control** |  |  |
| Chen 2022 | China | Retrospective cohort study | 26 | 28 | 30.9± 4.6 | 28.0 ± 4.3 | NR | NR | 4.0-10.0 mIU/L | Premature delivery, postpartum hemorrhage, low birth weight |
| Tu 2021 | China | Retrospective cohort study | 35 | 35 | 29.9 ± 2.3 | 29.8 ± 2.4 | 21.8 ± 1.1 | 21.7 ± 1.0 | > 4.0 mIU/L | Premature delivery, miscarriage, postpartum hemorrhage |
| Wang 2021 | China | RCT | 75 | 75 | 27.1 ± 2.9 | 26.9 ± 2.8 | 27.0 ± 2.4 | 26.9 ± 2.4 | > 4.0 mIU/L | Premature delivery, miscarriage, gestational diabetes, low birth weight |
| Li 2021 | China | Retrospective cohort study | 483 | 198 | NR | NR | NR | NR | > 4.0 mIU/L | Premature delivery, gestational hypertension, gestational diabetes, postpartum hemorrhage, placental abruption, premature rupture of membranes, fetal distress, low birth weight |
| Yoshihara 2021 | Japan | Prospective cohort study | 23 | 5 | 34.9 ± 4.3 | 34.3 ± 4.4 | NR | NR | 4.0-10.0 mIU/L | Miscarriage |
| Zhang 2020 | China | Retrospective cohort study | 115 | 48 | 28.5 ± 3.3 | 28.5 ± 3.3 | 26.8 ± 4.6 | 26.8 ± 4.6 | 4.0-10.0 mIU/L | Premature delivery, miscarriage, gestational hypertension, gestational diabetes, placental abruption, premature rupture of membranes, postpartum hemorrhage, small for gestational age |
| Zheng 2020 | China | RCT | 32 | 32 | 28.3 ± 1.3 | 27.9 ± 1.6 | NR | NR | 4.0-6.0 mIU/L | Premature delivery, miscarriage |
| Chen 2020 | China | Retrospective cohort study | 38 | 38 | 28.2 ± 4.4 | 28.4 ± 4.7 | NR | NR | > 4.0 mIU/L | Premature delivery, miscarriage, gestational hypertension, gestational diabetes, fetal growth restriction, low birth weight |
| He 2020 | China | Retrospective cohort study | 56 | 51 | 25.1 ± 2.8 | 25.5 ± 3.8 | 25.7 ± 4.6 | 25.4 ± 4.1 | > 4.0 mIU/L | Premature delivery, miscarriage |
| Lu 2019 | China | Prospective cohort study | 142 | 56 | 27.5 ± 4.1 | 26.8 ± 3.8 | NR | NR | > 97.5% | Premature delivery, miscarriage, gestational hypertension, gestational diabetes, postpartum hemorrhage |
| Shu 2019 | China | RCT | 67 | 67 | 29.7 ± 2.5 | 29.1 ± 2.0 | NR | NR | > 97.5% | Premature delivery, placental abruption, fetal distress, fetal growth restriction, low birth weight |
| Guo 2019 | China | Retrospective cohort study | 129 | 38 | 28.3 ± 2.3 | 28.9 ± 3.0 | NR | NR | 5.2-10.0 mIU/L | Children IQ, Childhood motor development |
| Chai 2018 | China | Retrospective cohort study | 2145 | 1367 | 30.0 ± 6.2 | 30.3 ± 5.3 | NR | NR | > 4.0 mIU/L | Premature delivery, miscarriage, gestational hypertension, placental abruption, fetal distress, fetal growth restriction, low birth weight |
| Nazarpour 2018 | Iran | RCT | 87 | 60 | 27.0 ± 5.3 | 26.9 ± 4.7 | 25.8 ± 5.0 | 26.0 ± 4.6 | > 4.0 mIU/L | Premature delivery |
| Nazarpour 2017 | Iran | RCT | 38 | 34 | 26.6 ± 5.8 | 27.0 ± 4.7 | 24.9 ± 5.1 | 24.6 ± 3.6 | > 4.0 mIU/L | Premature delivery, NICU admission |
| Casey 2017 | USA | RCT | 339 | 338 | 27.7 ± 5.7 | 27.3 ± 5.7 | 28.1 ± 6.4 | 28.2 ± 6.4 | > 4.0 mIU/L | Premature delivery, miscarriage, NICU admission, gestational hypertension, gestational diabetes, placental abruption, neonatal death, small for gestational age, respiratory distress syndrome, preeclampsia, Childhood IQ, Childhood behavioral and social competency |
| Maraka 2017 | USA | Retrospective cohort study | 513 | 697 | 31.7 ± 4.7 | 31.3 ± 5.2 | NR | NR | 4.1-10.0 mIU/L | Premature delivery, miscarriage, gestational hypertension, gestational diabetes, placental abruption, preeclampsia, fetal growth restriction |
| Xu 2016 | China | RCT | 30 | 30 | 28.1 ± 4.6 | 28.1 ± 4.6 | NR | NR | > 4.9 mIU/L | Premature delivery, miscarriage, gestational hypertension, gestational diabetes |
| Ju 2016 | China | Prospective cohort study | 184 | 273 | 29.3 ± 3.4 | 28.9 ± 3.5 | 24.7 ± 4.0 | 24.2 ± 3.6 | > 97.5% | Premature delivery, gestational hypertension, gestational diabetes, fetal distress, postpartum hemorrhage, low birth weight |
| Zhang 2016 | China | RCT | 570 | 570 | 24.6 ± 3.8 | 24.6 ± 3.8 | NR | NR | 5.2-10.0 mIU/L | Premature delivery, miscarriage, gestational hypertension, low birth weight |
| Yang 2015 | China | Retrospective cohort study | 1236 | 806 | NR | NR | NR | NR | 5.2-10.0 mIU/L | Premature delivery, miscarriage, gestational hypertension, gestational diabetes, placental abruption, fetal growth restriction, fetal distress, low birth weight |
| Kim 2011 | South Korea | RCT | 32 | 32 | 36.0 ± 2.4 | 36.1± 2.2 | 21.5 ± 1.9 | 21.7 ± 2.1 | > 4.5 mIU/L | miscarriage |

RCT, Randomized Controlled Trial; LT4, Levothyroxine; BMI, Body Mass Index; NICU, Neonatal intensive care unit; IQ, Intelligence Quotient; NR, not reported.

**Supplementary Table 3.** Risk of bias for each included cohort study.

| Study | Selection | | | | Comparability | Exposure | | | Score | Quality |
| --- | --- | --- | --- | --- | --- | --- | --- | --- | --- | --- |
|  | Representativ-eness of the exposed cohort | Selection of the non-exposed cohort | Ascertainment of exposure | Demonstration that outcome of interest was not present at start of study | Comparability of cohorts on the basis of the design or analysis | Assessment of outcome | Was follow-up long enough for outcomes to occur | Adequacy of follow-up of cohorts |  |  |
| Maraka 2017 | 1 | 0 | 1 | 1 | 2 | 1 | 1 | 0 | 7 | high |
| Yang 2015 | 0 | 0 | 0 | 0 | 0 | 1 | 1 | 0 | 2 | low |
| Guo 2019 | 0 | 0 | 0 | 1 | 0 | 1 | 1 | 0 | 3 | moderate |
| Li 2021 | 0 | 0 | 0 | 1 | 0 | 0 | 1 | 0 | 2 | low |
| Chen 2022 | 0 | 0 | 1 | 1 | 0 | 1 | 1 | 1 | 5 | moderate |
| Yoshihar 2021 | 0 | 0 | 0 | 1 | 2 | 1 | 1 | 0 | 5 | moderate |
| Ju 2016 | 0 | 1 | 0 | 1 | 2 | 0 | 1 | 0 | 5 | moderate |
| Chai 2018 | 0 | 0 | 0 | 1 | 0 | 0 | 1 | 0 | 2 | low |
| Chen 2020 | 0 | 0 | 0 | 0 | 0 | 0 | 1 | 0 | 1 | low |
| He 2020 | 0 | 1 | 0 | 0 | 0 | 0 | 1 | 0 | 2 | low |
| Lu 2019 | 0 | 0 | 0 | 0 | 0 | 0 | 1 | 0 | 1 | low |
| Tu 2021 | 0 | 0 | 0 | 0 | 0 | 0 | 1 | 0 | 1 | low |
| Zhang 2020 | 0 | 0 | 0 | 1 | 0 | 0 | 1 | 0 | 2 | low |

**Supplementary Table 4.** Meta-analysis results for secondary outcomes.

| Outcome | Study design | Numbers of studies | Numbers of patients | RR or OR (95%CI) | *P*-value | I2 | Model |
| --- | --- | --- | --- | --- | --- | --- | --- |
| Postpartum hemorrhage | all RCTs | 0 | 0 | NA | NA | NA | NA |
|  | RCTs with low and moderate risk of bias | 0 | 0 | NA | NA | NA | NA |
|  | all cohort studies | 6 | 1662 | 0.58 (0.40, 0.86) | 0.006 | 0% | Fix |
|  | cohort studies with low and moderate risk of bias | 2 | 550 | 1.05 (0.42, 2.66) | 0.910 | 0% | Fix |
| Placental abruption | all RCTs | 2 | 811 | 0.23 (0.04, 1.35) | 0.100 | 0% | Fix |
|  | RCTs with low and moderate risk of bias | 1 | 677 | 0.20 (0.02, 1.70) | 0.140 | 0% | Fix |
|  | all cohort studies | 4 | 6398 | 1.13 (0.73, 1.74) | 0.950 | 0% | Fix |
|  | cohort studies with low and moderate risk of bias | 0 | 0 | NA | NA | NA | NA |
| Fetal distress | all RCTs | 1 | 134 | 0.86 (0.30, 2.42) | 0.770 | 0% | Fix |
|  | RCTs with low and moderate risk of bias | 0 | 0 | NA | NA | NA | NA |
|  | all cohort studies | 5 | 6890 | 0.70 (0.55, 0.89) | 0.004 | 30% | Fix |
|  | cohort studies with low and moderate risk of bias | 1 | 457 | 0.78 (0.59, 1.04) | 0.090 | 0% | Fix |
| Premature rupture of membranes | all RCTs | 0 | 0 | NA | NA | NA | NA |
|  | RCTs with low and moderate risk of bias | 0 | 0 | NA | NA | NA | NA |
|  | all cohort studies | 2 | 1138 | 0.73 (0.51, 1.05) | 0.090 | 0% | Fix |
|  | cohort studies with low and moderate risk of bias | 1 | 457 | 0.73 (0.48, 1.10) | 0.130 | 0% | Fix |
| Fetal growth restriction | all RCTs | 1 | 134 | 0.50 (0.16, 1.58) | 0.240 | 0% | Fix |
|  | RCTs with low and moderate risk of bias | 0 | 0 | NA | NA | NA | NA |
|  | all cohort studies | 4 | 6840 | 0.79 (0.60, 1.03) | 0.080 | 51% | Random |
|  | cohort studies with low and moderate risk of bias | 1 | 1210 | 1.17 (0.79, 1.73) | 0.430 | 0% | Fix |
| Low birth weight | all RCTs | 2 | 1274 | 0.45 (0.19, 1.08) | 0.070 | 54% | Random |
|  | RCTs with low and moderate risk of bias | 0 | 0 | NA | NA | NA | NA |
|  | all cohort studies | 8 | 7166 | 0.73 (0.61, 0.87) | < 0.001 | 15% | Fix |
|  | cohort studies with low and moderate risk of bias | 2 | 550 | 1.47 (0.53, 4.11) | 0.46 | 0% | Fix |
| Small for gestational age | all RCTs | 1 | 667 | 1.22 (0.75, 1.98) | 0.430 | 0% | Fix |
|  | RCTs with low and moderate risk of bias | 1 | 667 | 1.22 (0.75, 1.98) | 0.430 | 0% | Fix |
|  | all cohort studies | 1 | 163 | 2.14 (0.24, 18.79) | 0.490 | 0% | Fix |
|  | cohort studies with low and moderate risk of bias | 0 | 0 | NA | NA | NA | NA |
| NICU admission | all RCTs | 2 | 749 | 0.31 (0.01, 12.16) | 0.530 | 85% | Random |
|  | RCTs with low and moderate risk of bias | 2 | 749 | 0.31 (0.01, 12.16) | 0.530 | 85% | Random |
|  | all cohort studies | 0 | 0 | NA | NA | NA | NA |
|  | cohort studies with low and moderate risk of bias | 0 | 0 | NA | NA | NA | NA |
| Neonatal death | all RCTs | 1 | 677 | 0.33 (0.01, 8.13) | 0.500 | 0% | Fix |
|  | RCTs with low and moderate risk of bias | 1 | 677 | 0.33 (0.01, 8.13) | 0.500 | 0% | Fix |
|  | all cohort studies | 0 | 0 | NA | NA | NA | NA |
|  | cohort studies with low and moderate risk of bias | 0 | 0 | NA | NA | NA | NA |
| Respiratory distress syndrome | all RCTs | 1 | 677 | 1.50 (0.54, 4.16) | 0.440 | 0% | Fix |
|  | RCTs with low and moderate risk of bias | 1 | 677 | 1.50 (0.54, 4.16) | 0.440 | 0% | Fix |
|  | all cohort studies | 0 | 0 | NA | NA | NA | NA |
|  | cohort studies with low and moderate risk of bias | 0 | 0 | NA | NA | NA | NA |

RCTs, randomized controlled trials; RR, relative risk; OR, odds ratio; NICU, neonatal intensive care unit; CI, confidence interval; I^2^, statistical heterogeneity; NA, not applicable since no studies were included; According to the pre-defined rules, the meta-analysis results with gray background were used to draw conclusions for each outcome.

**Supplementary Table 5.** Grade evidence profile for each outcome.

| **Quality assessment** | | | | | | | **No of patients** | | **Effect** | | **Quality** | **Importance** | |  |
| --- | --- | --- | --- | --- | --- | --- | --- | --- | --- | --- | --- | --- | --- | --- |
|  |  |  |  |  |  |  |  |  |  |  |  |  |  |  |
| **No of studies** | **Design** | **Risk of bias** | **Inconsistency** | **Indirectness** | **Imprecision** | **Other considerations** | **LT4** | **Control** | **Relative (95% CI)** | **Absolute** |  |  |  |  |
| **Preterm delivery** | | | | | | | | | | | | | |  |
| 3 | randomised trials | no serious risk of bias | serious^1^ | no serious indirectness | serious^2^ | none | 39/464  (8.4%) | 58/431  (13.5%) | RR 0.62 (0.42 to 0.91) | 51 fewer per 1000 (from 12 fewer to 78 fewer) | ⊕⊕OO LOW | CRITICAL | |  |
|  |  |  |  |  |  |  |  | 18.6% |  | 71 fewer per 1000 (from 17 fewer to 108 fewer) |  |  |  |  |
| **Miscarriage** | | | | | | | | | | | | | |  |
| 2 | randomised trials | no serious risk of bias | no serious inconsistency | no serious indirectness | Serious^2^ | none | 4/356  (1.1%) | 11/350  (3.1%) | RR 0.36 (0.13 to 1.03) | 20 fewer per 1000 (from 27 fewer to 1 more) | ⊕⊕⊕O MODERATE | | CRITICAL | |
|  |  |  |  |  |  |  |  | 17.7% |  | 113 fewer per 1000 (from 154 fewer to 5 more) |  |  |  |  |
| **Gestational hypertension** | | | | | | | | | | | | | |  |
| 1 | randomised trials | no serious risk of bias | no serious inconsistency | no serious indirectness | serious^2^ | none | 33/339  (9.7%) | 36/338  (10.7%) | RR 0.91 (0.58 to 1.43) | 10 fewer per 1000 (from 45 fewer to 46 more) | ⊕⊕⊕O MODERATE | | IMPORTANT | |
|  |  |  |  |  |  |  |  | 10.7% |  | 10 fewer per 1000 (from 45 fewer to 46 more) |  |  |  |  |
| **Gestational diabetes** | | | | | | | | | | | | | |  |
| 4 | randomised trials | no serious risk of bias | no serious inconsistency | no serious indirectness | serious^2^ | none | 31/511  (6.1%) | 39/510  (7.6%) | RR 0.8 (0.51 to 1.25) | 15 fewer per 1000 (from 37 fewer to 19 more) | ⊕⊕⊕O MODERATE | | IMPORTANT | |
|  |  |  |  |  |  |  |  | 5.3% |  | 11 fewer per 1000 (from 26 fewer to 13 more) |  |  |  |  |
| **Postpartum hemorrhage** | | | | | | | | | | | | | |  |
| 2 | observational studies | no serious risk of bias | no serious inconsistency | no serious indirectness | very serious^2^ | none | 8/225  (3.6%) | 11/325  (3.4%) | OR 1.05 (0.42 to 2.66) | 2 more per 1000 (from 19 fewer to 51 more) | ⊕OOO VERY LOW | | IMPORTANT | |
|  |  |  |  |  |  |  |  | 3.6% |  | 2 more per 1000 (from 21 fewer to 54 more) |  |  |  |  |
| **Preeclampsia** | | | | | | | | | | | | | |  |
| 1 | randomised trials | no serious risk of bias | no serious inconsistency | no serious indirectness | very serious^2^ | none | 22/339  (6.5%) | 20/338  (5.9%) | RR 1.1 (0.61 to 1.97) | 6 more per 1000 (from 23 fewer to 57 more) | ⊕⊕OO LOW | | IMPORTANT | |
|  |  |  |  |  |  |  |  | 5.9% |  | 6 more per 1000 (from 23 fewer to 57 more) |  |  |  |  |
| **Placental abruption** | | | | | | | | | | | | | |  |
| 2 | randomised trials | no serious risk of bias | no serious inconsistency | no serious indirectness | very serious^2^ | none | 1/406  (0.2%) | 6/405  (1.5%) | RR 0.23 (0.04 to 1.35) | 11 fewer per 1000 (from 14 fewer to 5 more) | ⊕⊕OO LOW | | IMPORTANT | |
|  |  |  |  |  |  |  |  | 1.5% |  | 12 fewer per 1000 (from 14 fewer to 5 more) |  |  |  |  |
| **Fetal growth restriction** | | | | | | | | | | | | | |  |
| 1 | randomised trials | Serious^3^ | no serious inconsistency | no serious indirectness | very serious^2^ | reporting bias^4^ | 4/67  (6%) | 8/67  (11.9%) | RR 0.5 (0.16 to 1.58) | 60 fewer per 1000 (from 100 fewer to 69 more) | ⊕OOO VERY LOW | | IMPORTANT | |
|  |  |  |  |  |  |  |  | 11.9% |  | 59 fewer per 1000 (from 100 fewer to 69 more) |  |  |  |  |
| **Fetal distress** | | | | | | | | | | | | | |  |
| 1 | randomised trials | Serious^3^ | no serious inconsistency | no serious indirectness | very serious^2^ | reporting bias^4^ | 6/67  (9%) | 7/67  (10.4%) | RR 0.86 (0.3 to 2.42) | 15 fewer per 1000 (from 73 fewer to 148 more) | ⊕OOO VERY LOW | | IMPORTANT | |
|  |  |  |  |  |  |  |  | 10.5% |  | 15 fewer per 1000 (from 73 fewer to 149 more) |  |  |  |  |
| **Premature rupture of membranes** | | | | | | | | | | | | | |  |
| 2 | observational studies | no serious risk of bias | no serious inconsistency | no serious indirectness | Serious^2^ | none | 72/667  (10.8%) | 79/471  (16.8%) | OR 0.73 (0.51 to 1.05) | 39 fewer per 1000 (from 75 fewer to 7 more) | ⊕OOO VERY LOW | | IMPORTANT | |
|  |  |  |  |  |  |  |  | 16% |  | 38 fewer per 1000 (from 71 fewer to 7 more) |  |  |  |  |
| **Low birth weight** | | | | | | | | | | | | | |  |
| 2 | randomised trials | Serious^5^ | Serious^1^ | Serious^6^ | Serious^2^ | none | 25/637  (3.9%) | 67/637  (10.5%) | RR 0.45 (0.19 to 1.08) | 58 fewer per 1000 (from 85 fewer to 8 more) | ⊕OOO VERY LOW | | IMPORTANT | |
|  |  |  |  |  |  |  |  | 9.8% |  | 54 fewer per 1000 (from 79 fewer to 8 more) |  |  |  |  |
| **Small for gestational age** | | | | | | | | | | | | | |  |
| 1 | randomised trials | no serious risk of bias | no serious inconsistency | no serious indirectness | Serious^2^ | none | 33/339  (9.7%) | 27/338  (8%) | RR 1.22 (0.75 to 1.98) | 18 more per 1000 (from 20 fewer to 78 more) | ⊕⊕⊕O MODERATE | | IMPORTANT | |
|  |  |  |  |  |  |  |  | 8% |  | 18 more per 1000 (from 20 fewer to 78 more) |  |  |  |  |
| **NICU admission** | | | | | | | | | | | | | |  |
| 2 | randomised trials | no serious risk of bias | very serious^1^ | no serious indirectness | very serious^2^ | none | 29/377  (7.7%) | 31/372  (8.3%) | RR 0.31 (0.01 to 12.16) | 57 fewer per 1000 (from 82 fewer to 930 more) | ⊕OOO VERY LOW | | IMPORTANT | |
|  |  |  |  |  |  |  |  | 17.8% |  | 123 fewer per 1000 (from 176 fewer to 1000 more) |  |  |  |  |
| **Neonatal death** | | | | | | | | | | | | | |  |
| 1 | randomised trials | no serious risk of bias | no serious inconsistency | no serious indirectness | very serious^2^ | none | 0/339  (0%) | 1/338  (0.3%) | RR 0.33 (0.01 to 8.13) | 2 fewer per 1000 (from 3 fewer to 21 more) | ⊕⊕OO LOW | | CRITICAL | |
|  |  |  |  |  |  |  |  | 0.3% |  | 2 fewer per 1000 (from 3 fewer to 21 more) |  |  |  |  |
| **Respiratory distress syndrome** | | | | | | | | | | | | | |  |
| 1 | randomised trials | no serious risk of bias | no serious inconsistency | no serious indirectness | very serious^2^ | none | 9/339  (2.7%) | 6/338  (1.8%) | RR 1.5 (0.54 to 4.16) | 9 more per 1000 (from 8 fewer to 56 more) | ⊕⊕OO LOW | | IMPORTANT | |
|  |  |  |  |  |  |  |  | 1.8% |  | 9 more per 1000 (from 8 fewer to 57 more) |  |  |  |  |

CI, confidence interval; RR, risk ratio; OR, odds ratio; NICU, neonatal intensive care unit.

^1^ Significant heterogeneity

^2^ 95% confidence interval was wide

^3^ Only one study was included and the risk of bias was high

^4^ The number of included studies was small and the sample size was small

^5^ All included studies had a high risk of bias

^6^ There are some differences in the study population

**Supplementary Table 6.** Grade evidence profile for each outcome in the TPOAb-positive subgroup.

| **Quality assessment** | | | | | | | **No of patients** | | **Effect** | | **Quality** | **Importance** |
| --- | --- | --- | --- | --- | --- | --- | --- | --- | --- | --- | --- | --- |
|  |  |  |  |  |  |  |  |  |  |  |  |  |
| **No of studies** | **Design** | **Risk of bias** | **Inconsistency** | **Indirectness** | **Imprecision** | **Other considerations** | **LT4** | **Control** | **Relative (95% CI)** | **Absolute** |  |  |
| Preterm delivery | | | | | | | | | | | | |
| 1 | randomised trials | no serious risk of bias | no serious inconsistency | no serious indirectness | serious^1^ | reporting bias^2^ | 2/38  (5.3%) | 10/34  (29.4%) | RR 0.18 (0.04 to 0.76) | 241 fewer per 1000 (from 71 fewer to 282 fewer) | ⊕⊕OO LOW | CRITICAL |
|  |  |  |  |  |  |  |  | 29.4% |  | 241 fewer per 1000 (from 71 fewer to 282 fewer) |  |  |
| Miscarriage | | | | | | | | | | | | |
| 3 | observational studies | very serious^3^ | no serious inconsistency | no serious indirectness | no serious imprecision | strong association^4^ | 112/2831  (4%) | 65/580  (11.2%) | OR 0.32 (0.23 to 0.44) | 73 fewer per 1000 (from 59 fewer to 84 fewer) | ⊕OOO VERY LOW | CRITICAL |
|  |  |  |  |  |  |  |  | 11.4% |  | 74 fewer per 1000 (from 60 fewer to 85 fewer) |  |  |
| Gestational hypertension | | | | | | | | | | | | |
| 3 | observational studies | Serious^3^ | Serious^5^ | no serious indirectness | no serious imprecision | strong association^4^ | 301/2831  (10.6%) | 168/580  (29%) | OR 0.29 (0.17 to 0.5) | 184 fewer per 1000 (from 120 fewer to 225 fewer) | ⊕OOO VERY LOW | IMPORTANT |
|  |  |  |  |  |  |  |  | 22.9% |  | 150 fewer per 1000 (from 100 fewer to 181 fewer) |  |  |
| Gestational diabetes | | | | | | | | | | | | |
| 2 | observational studies | Serious^3^ | no serious inconsistency | no serious indirectness | Serious^1^ | strong association^4^ | 43/1059  (4.1%) | 21/239  (8.8%) | OR 0.43 (0.25 to 0.76) | 48 fewer per 1000 (from 20 fewer to 64 fewer) | ⊕OOO VERY LOW | IMPORTANT |
|  |  |  |  |  |  |  |  | 9.5% |  | 52 fewer per 1000 (from 21 fewer to 69 fewer) |  |  |
| Placental abruption | | | | | | | | | | | | |
| 2 | observational studies | Serious^3^ | no serious inconsistency | no serious indirectness | very serious^1^ | none | 26/2793  (0.9%) | 6/542  (1.1%) | OR 0.84 (0.34 to 2.06) | 2 fewer per 1000 (from 7 fewer to 11 more) | ⊕OOO VERY LOW | IMPORTANT |
|  |  |  |  |  |  |  |  | 1.2% |  | 2 fewer per 1000 (from 8 fewer to 12 more) |  |  |
| Fetal growth restriction | | | | | | | | | | | | |
| 3 | observational studies | Serious^3^ | no serious inconsistency | no serious indirectness | no serious imprecision | reporting bias strong association^4^ | 245/2831  (8.7%) | 111/580  (19.1%) | OR 0.38 (0.3 to 0.49) | 109 fewer per 1000 (from 87 fewer to 125 fewer) | ⊕OOO VERY LOW | IMPORTANT |
|  |  |  |  |  |  |  |  | 19.9% |  | 113 fewer per 1000 (from 90 fewer to 130 fewer) |  |  |
| Fetal distress | | | | | | | | | | | | |
| 2 | observational studies | Serious^3^ | no serious inconsistency | no serious indirectness | very serious^1^ | none | 46/2793  (1.6%) | 8/542  (1.5%) | OR 1.12 (0.52 to 2.38) | 2 more per 1000 (from 7 fewer to 20 more) | ⊕OOO VERY LOW | CRITICAL |
|  |  |  |  |  |  |  |  | 1.5% |  | 2 more per 1000 (from 7 fewer to 20 more) |  |  |
| Low birth weight | | | | | | | | | | | | |
| 3 | observational studies | very serious^3^ | no serious inconsistency | no serious indirectness | no serious imprecision | strong association^4^ | 205/2831  (7.2%) | 90/580  (15.5%) | OR 0.4 (0.31 to 0.53) | 87 fewer per 1000 (from 66 fewer to 101 fewer) | ⊕OOO VERY LOW | IMPORTANT |
|  |  |  |  |  |  |  |  | 16.4% |  | 91 fewer per 1000 (from 70 fewer to 107 fewer) |  |  |
| NICU admission | | | | | | | | | | | | |
| 1 | randomised trials | no serious risk of bias | no serious inconsistency | no serious indirectness | Serious^6^ | reporting bias^2^ | 0/38  (0%) | 10/34  (29.4%) | RR 0.04 (0 to 0.7) | 282 fewer per 1000 (from 88 fewer to 294 fewer) | ⊕⊕OO LOW | IMPORTANT |
|  |  |  |  |  |  |  |  | 29.4% |  | 282 fewer per 1000 (from 88 fewer to 294 fewer) |  |  |

CI, confidence interval; RR, risk ratio; OR, odds ratio; NICU, neonatal intensive care unit.

^1^95% confidence interval was wide

^2^ The number of included studies was small

^3^ All included studies had a high risk of bias

^4^ Large effect value

^5^ Significant heterogeneitise

^6^ The sample size was small

**Supplementary Table 7.** Grade evidence profile for each outcome in the TPOAb-negative subgroup.

| **Quality assessment** | | | | | | | | **No of patients** | | | | **Effect** | | **Quality** | **Importance** |
| --- | --- | --- | --- | --- | --- | --- | --- | --- | --- | --- | --- | --- | --- | --- | --- |
|  |  |  |  |  |  |  |  |  |  |  |  |  |  |  |  |
| **No of studies** | **Design** | **Risk of bias** | **Inconsistency** | | **Indirectness** | **Imprecision** | **Other considerations** | **LT4** | **Control** | | | **Relative (95% CI)** | **Absolute** |  |  |
| **Preterm delivery** | | | | | | | | | | | | | | | |
| 4 | randomised trials | serious^1^ | no serious inconsistency | | no serious indirectness | no serious imprecision | reporting bias^2^ | 44/754  (5.8%) | 86/726  (11.8%) | | | RR 0.49 (0.34 to 0.69) | 60 fewer per 1000 (from 37 fewer to 78 fewer) | ⊕⊕OO LOW | CRITICAL |
|  |  |  |  |  |  |  |  |  | 17.5% | | |  | 89 fewer per 1000 (from 54 fewer to 115 fewer) |  |  |
| **Miscarriage** | | | | | | | | | | | | | | | |
| 3 | randomised trials | very serious^3^ | no serious inconsistency | | no serious indirectness | no serious imprecision | none | 51/667  (7.6%) | 119/667  (17.8%) | | | RR 0.43 (0.32 to 0.59) | 102 fewer per 1000 (from 73 fewer to 121 fewer) | ⊕⊕OO LOW | CRITICAL |
|  |  |  |  |  |  |  |  |  | 17.9% | | |  | 102 fewer per 1000 (from 73 fewer to 122 fewer) |  |  |
| **Gestational hypertension** | | | | | | | | | | | | | | | |
| 3 | randomised trials | very serious^3^ | no serious inconsistency | | no serious indirectness | no serious imprecision | none | 37/667  (5.5%) | 75/667  (11.2%) | | | RR 0.49 (0.34 to 0.72) | 57 fewer per 1000 (from 31 fewer to 74 fewer) | ⊕⊕OO LOW | IMPORTANT |
|  |  |  |  |  |  |  |  |  | 13.4% | | |  | 68 fewer per 1000 (from 38 fewer to 88 fewer) |  |  |
| **Gestational diabetes** | | | | | | | | | | | | | | | |
| 2 | randomised trials | very serious^3^ | no serious inconsistency | | no serious indirectness | Serious^4^ | None^5^ | 5/97  (5.2%) | 14/97  (14.4%) | | | RR 0.38 (0.15 to 0.96) | 89 fewer per 1000 (from 6 fewer to 123 fewer) | ⊕OOO VERY LOW | IMPORTANT |
|  |  |  |  |  |  |  |  |  | 11.4% | | |  | 71 fewer per 1000 (from 5 fewer to 97 fewer) |  |  |
| **Postpartum hemorrhage** | | | | | | | | | | | | | | | |
| 1 | observational studies | no serious risk of bias | no serious inconsistency | | no serious indirectness | very serious^4^ | reporting bias^5^ | 1/41  (2.4%) | | 2/52  (3.8%) | | OR 0.62 (0.05 to 7.14) | 14 fewer per 1000 (from 36 fewer to 184 more) | ⊕OOO VERY LOW | IMPORTANT |
|  |  |  |  |  |  |  |  |  | | 3.9% | |  | 14 fewer per 1000 (from 37 fewer to 186 more) |  |  |
| **Placental abruption** | | | | | | | | | | | | | | | |
| 1 | randomised trials | Serious^6^ | no serious inconsistency | | no serious indirectness | very serious^4^ | reporting bias^5^ | 0/67  (0%) | | 1/67  (1.5%) | | RR 0.33 (0.01 to 8.04) | 10 fewer per 1000 (from 15 fewer to 105 more) | ⊕OOO VERY LOW | IMPORTANT |
|  |  |  |  |  |  |  |  |  | | 1.5% | |  | 10 fewer per 1000 (from 15 fewer to 106 more) |  |  |
| **Fetal growth restriction** | | | | | | | | | | | | | | | |
| 1 | randomised trials | Serious^6^ | no serious inconsistency | | no serious indirectness | Serious^4^ | reporting bias^5^ | 4/67  (6%) | | | 8/67  (11.9%) | RR 0.5 (0.16 to 1.58) | 60 fewer per 1000 (from 100 fewer to 69 more) | ⊕OOO VERY LOW | IMPORTANT |
|  |  |  |  |  |  |  |  |  | | | 11.9% |  | 59 fewer per 1000 (from 100 fewer to 69 more) |  |  |
| **Fetal distress** | | | | | | | | | | | | | | | |
| 1 | randomised trials | Serious^6^ | no serious inconsistency | | no serious indirectness | very serious^4^ | reporting bias^5^ | 6/67  (9%) | | 7/67  (10.4%) | | RR 0.86 (0.3 to 2.42) | 15 fewer per 1000 (from 73 fewer to 148 more) | ⊕OOO VERY LOW | CRITICAL |
|  |  |  |  |  |  |  |  |  | | 10.5% | |  | 15 fewer per 1000 (from 73 fewer to 149 more) |  |  |
| **Premature rupture of membranes** | | | | | | | | | | | | | | | |
| 1 | observational studies | Serious^6^ | no serious inconsistency | no serious indirectness | | very serious^4^ | none | 44/483  (9.1%) | | 22/198  (11.1%) | | OR 0.8 (0.47 to 1.38) | 20 fewer per 1000 (from 56 fewer to 36 more) | ⊕OOO VERY LOW | IMPORTANT |
|  |  |  |  |  |  |  |  |  | | 11.1% | |  | 20 fewer per 1000 (from 56 fewer to 36 more) |  |  |
| **Low birth weight** | | | | | | | | | | | | | | | |
| 2 | randomised trials | serious^1^ | serious^2^ | no serious indirectness | | Serious^4^ | none | 25/637  (3.9%) | | 67/637  (10.5%) | | RR 0.45 (0.19 to 1.08) | 58 fewer per 1000 (from 85 fewer to 8 more) | ⊕OOO VERY LOW | IMPORTANT |
|  |  |  |  |  |  |  |  |  | | 9.8% | |  | 54 fewer per 1000 (from 79 fewer to 8 more) |  |  |

CI, confidence interval; RR, risk ratio; OR, odds ratio.

^1^ Three quarters of the studies had a high risk of bias

^2^ Small number of studies

^3^ All included studies had a high risk of bias

^4^ 95% confidence interval was wide

^5^ The number of included studies was small and the sample size was small

^6^ Only one study was included and the risk of bias was high
